# Phosphorylation Regulates CIRBP Arginine Methylation, Transportin-1 Binding and Liquid-Liquid Phase Separation

**DOI:** 10.3389/fmolb.2021.689687

**Published:** 2021-10-19

**Authors:** Aneta J. Lenard, Saskia Hutten, Qishun Zhou, Sinem Usluer, Fangrong Zhang, Benjamin M. R. Bourgeois, Dorothee Dormann, Tobias Madl

**Affiliations:** ^1^ Gottfried Schatz Research Center for Cell Signaling, Metabolism and Aging, Molecular Biology and Biochemistry, Medical University of Graz, Graz, Austria; ^2^ Johannes Gutenberg-Universität (JGU) Mainz, Faculty of Biology, Mainz, Germany; ^3^ BioMedical Center, Cell Biology, Ludwig-Maximilians-Universität (LMU) München, Martinsried, Germany; ^4^ Institute of Molecular Biology (IMB), Mainz, Germany; ^5^ Munich Cluster for Systems Neurology (SyNergy), Munich, Germany; ^6^ BioTechMed-Graz, Graz, Austria

**Keywords:** RNA-binding proteins, CIRBP, SRPK1, phosphorylation, arginine methylation, PTMs, liquid-liquid phase separation, transportin-1

## Abstract

Arginine-glycine(-glycine) (RG/RGG) regions are highly abundant in RNA-binding proteins and involved in numerous physiological processes. Aberrant liquid-liquid phase separation (LLPS) and stress granule (SGs) association of RG/RGG regions in the cytoplasm have been implicated in several neurodegenerative disorders. LLPS and SG association of these proteins is regulated by the interaction with nuclear import receptors, such as transportin-1 (TNPO1), and by post-translational arginine methylation. Strikingly, many RG/RGG proteins harbour potential phosphorylation sites within or close to their arginine methylated regions, indicating a regulatory role. Here, we studied the role of phosphorylation within RG/RGG regions on arginine methylation, TNPO1-binding and LLPS using the cold-inducible RNA-binding protein (CIRBP) as a paradigm. We show that the RG/RGG region of CIRBP is *in vitro* phosphorylated by serine-arginine protein kinase 1 (SRPK1), and discovered two novel phosphorylation sites in CIRBP. SRPK1-mediated phosphorylation of the CIRBP RG/RGG region impairs LLPS and binding to TNPO1 *in vitro* and interferes with SG association in cells. Furthermore, we uncovered that arginine methylation of the CIRBP RG/RGG region regulates *in vitro* phosphorylation by SRPK1. In conclusion, our findings indicate that LLPS and TNPO1-mediated chaperoning of RG/RGG proteins is regulated through an intricate interplay of post-translational modifications.

## Introduction

A growing number of evidences has emerged over the last decade implicating that cells organize a plethora of biochemical processes by means of biomolecular condensation, including the formation of membraneless ribonucleoprotein (RNP) granules (Banani et al., 2016; Banani et al., 2017; Shin and Brangwynne, 2017). RNP granules constitute micron-sized, condensed, dynamic assemblies of RNA and RNA-binding proteins (RBPs), exemplified by nucleoli, Cajal bodies, paraspeckles in the nucleus or stress granules (SGs), and P-bodies in the cytoplasm (Hyman et al., 2014; Molliex et al., 2015; Feric et al., 2016). These membraneless organelles are proposed to form through the process of liquid-liquid phase separation (LLPS), by which coexisting protein/RNA-depleted (dilute) and highly protein/RNA-enriched (condensed) phases emerge and remain in a dynamic equilibrium with the cellular surroundings (Aguzzi and Altmeyer, 2016; Alberti, 2017; Ditlev et al., 2018; Alberti et al., 2019). Multivalent interactions have been recognized as a critical factor driving the assembly of protein/RNA into the condensed phase. They are facilitated by the modular structure of RBPs possessing multiple RNA-binding domains and intrinsically disordered regions with low complexity sequences of amino acids (Li et al., 2012; Banani et al., 2017; Chong et al., 2018; Martin and Holehouse, 2020). Importantly, RNP compartments and missense mutations in RBPs are thought to be central to the pathogenesis of several neuronal disorders such as amyotrophic lateral sclerosis (ALS), frontotemporal dementia (FTD), inclusion body myopathy (IBM) (Li et al., 2013; Ramaswami et al., 2013; Tsang et al., 2020). These diseases are characterized by the presence of mis-localized pathological protein aggregates formed in the cytoplasm of neuronal cells, and till now no effective therapies targeting them have been reported (Harrison and Shorter, 2017).

Proteins containing RG/RGG-regions are highly abundant in the eukaryotic proteome, and have been demonstrated to localize to cellular RNP granules (Thandapani et al., 2013). For example, SGs contain a large number of RG/RGG proteins, e.g. members of FET protein family (including FUS, EWS and TAF15) (Andersson et al., 2008; Dormann et al., 2010), TDP-43 (Bentmann et al., 2012), FMRP (Didiot et al., 2009), G3BP1 (Tourrière et al., 2003) and CAPRIN-1 (Solomon et al., 2007), nucleoli contain nucleolin and fibrillarin (Frottin et al., 2019), the RG-dipeptide repeats containing coilin is a marker for Cajal bodies (Hebert et al., 2002), and Lsm14a can be found in P-bodies (Yang et al., 2006). Purified proteins containing RG/RGG-regions have been shown to undergo LLPS *in vitro* in a reversible and concentration-dependent manner, and that addition of RNA can enhance their propensity for phase separation (Patel et al., 2015; Boeynaems et al., 2017; Chong et al., 2018). Wang *et al.* determined a sequence-encoded “molecular grammar” where the interactions between aromatic and positively charged residues have been identified as critical for phase separation of RBPs (Wang et al., 2018), and various studies showed that arginines are necessary for LLPS of RG/RGG regions- or RG-FG repeats-containing proteins (Elbaum-Garfinkle et al., 2015; Nott et al., 2015; Hofweber et al., 2018; Yang et al., 2020). Moreover, post-translational modifications (PTMs) within RG/RGG regions provide a means of phase separation regulation (Chong et al., 2018; Rhoads et al., 2018; Hofweber and Dormann, 2019). For instance, methylation of arginines in FUS, hnRNP-A2, FMRP, and DDX4 suppresses their LLPS by reducing arginine-(pi) aromatic interactions (Nott et al., 2015; Hofweber et al., 2018; Qamar et al., 2018; Ryan et al., 2018; Tsang et al., 2019). In addition, arginine methylation impairs SG association of RG/RGG proteins, such as G3BP1 (Tsai et al., 2016), FUS (Hofweber et al., 2018), FMRP (Dolzhanskaya et al., 2006), CIRBP (De Leeuw et al., 2007).

Another PTM frequently occurring in RBPs is phosphorylation of serine (in mammals ∼90% of phosphorylation events occur on serines), threonine, or tyrosine residues, which introduces a double negative charge via a phosphate group (Bah and Forman-Kay, 2016). In contrast to arginine methylation, phosphorylation can regulate LLPS of RBPs either positively or negatively (Wang et al., 2018; Hofweber and Dormann, 2019). For example, phosphorylation of the low-complexity domain of FUS disrupts *in vitro* phase separation (Monahan et al., 2017), whereas phosphorylation within the low-complexity region of FMRP promotes LLPS *in vitro* (Tsang et al., 2019). Phosphorylation of G3BP1 on serine-149 by casein kinase 2 (CK2) as well as dual specificity tyrosine phosphorylation–regulated kinase 3 (DYRK3) – mediated phosphorylation of multiple RBPs have been shown to disassemble corresponding membraneless organelles (Wippich et al., 2013; Reineke et al., 2018). On the contrary, SG localization of 5′-AMP-activated protein kinase-α2 (AMPK-α2) and mTOR (mechanistic target of rapamycin) effector kinases S6 kinase 1 and 2 (S6K1 and S6K2) are required for SG assembly (Mahboubi et al., 2015; Sfakianos et al., 2018).

RBPs often carry a combination of multiple PTMs, in which modifications can affect one another when located closely in the primary sequence or 3D space (PTM cross-talk). For instance, a recent study conducted a bioinformatic analysis focused on SRGG motifs (overlapping SR and RGG regions, with serine serving as a site for phosphorylation, and arginine as a site for methylation) in the *S. cerevisiae* proteome (Smith et al., 2020). The authors identified 38 yeast proteins harboring the SRGG motif, and only three of them – Nop1p, Npl3p, and Gar1p – possess multiple repeats of the SRGG region. They further demonstrated for Nop1p that the presence of serine phosphorylation within the SRGG motif blocks arginine methylation by a yeast methyltransferase within the same and adjacent motifs, as well as that the presence of arginine methylation in the SRGG region decreases serine phosphorylation. Besides arginine methylation and phosphorylation, many other PTMs appear in RBPs and may affect their LLPS, as exemplified by arginine-to-citrulline conversion (Tanikawa et al., 2018), lysine acetylation (Saito et al., 2019), or *O-*GlcNAcylation (Ohn et al., 2008). Thus, further studies are needed to fill gaps in our knowledge about the crosstalk between PTMs as well as the impact of various modifications on LLPS.

In addition to aberrant arginine methylation, defective nucleocytoplasmic transport of RBPs is a crucial pathological factor driving the onset of ALS/FTD disorders (Dormann et al., 2010). We and others have previously reported that the nuclear import receptor Transportin-1 (TNPO1)/Karyopherin-β2 (Kapβ2) acts as a chaperone for the RBP FUS, and reduces both its phase separation and SG recruitment via direct interaction with the RGG3-PY (proline-tyrosine) region of FUS (Guo et al., 2018; Hofweber et al., 2018; Qamar et al., 2018; Yoshizawa et al., 2018). Nucleocytoplasmic shuttling and chaperoning activity of importins are believed to be dependent on the specific interaction between an importin and a nuclear localization signal (NLS) within its cargo protein (Chook and Blobel, 2001; Soniat and Chook, 2015; Frey et al., 2018). Recently, we have identified the RG/RGG region and an arginine-serine-tyrosine (RSY)–rich region in cold-inducible RNA-binding protein (CIRBP) to serve as NLSs for transportin-1 and transportin-3 (Bourgeois et al., 2020). CIRBP is a member of the family of cold shock proteins. In response to different cellular stresses, such as mild cold shock, ultraviolet irradiation, osmotic shock, or hypoxia, CIRBP relocalizes from the nucleus to the cytoplasm where it partitions into SGs (Aoki et al., 2002; Pan et al., 2004; De Leeuw et al., 2007). CIRBP plays anti-apoptotic and anti-senescent roles in cells (Sakurai et al., 2006; Lee et al., 2015a), and its mis-regulation is associated with numerous pathologies. CIRBP is involved in the development of brain ischemia (Zhou et al., 2014), and extracellular CIRBP triggers inflammation and tissue injury in sepsis by inducing the formation of neutrophil extracellular traps in patients lungs (Ode et al., 2018; Ode et al., 2019). Furthermore, CIRBP constitutes a promising target for anticancer therapy, as its downregulation was found to inhibit cancer cell survival in patients suffering from liver, breast, brain, and prostate cancers (Zeng et al., 2009; Lujan et al., 2018).

Despite our growing understanding of liquid-liquid phase transition phenomena in living cells, we still lack of a full comprehension of their regulation, for example how LLPS of RBPs is regulated. In this study, we show that the RG/RGG region of CIRBP (CIRBP-RGG) is phosphorylated in cell lysate and identified serine-arginine protein kinase-1 (SRPK1) as a relevant kinase *in vitro*. Phosphorylation of CIRBP-RGG inhibited methylation of adjacent arginines and vice versa. *In vitro*, SRPK1-mediated phosphorylation of CIRBP-RGG suppresses phase separation, and in semi-permeabilized cells, it suppresses SG recruitment of CIRBP. Our study furthermore reveals that phosphorylation of CIRBP-RGG impairs binding to the nuclear import receptor Transportin-1 (TNPO1). Summarizing, our study sheds light on the regulation of membraneless organelles and nuclear translocation of RG/RGG region-containing proteins via an intricate interplay of PTMs.

## Materials and Methods

### Recombinant Protein Expression and Purification

Recombinant His_6_-protein A-tagged CIRBP-RGG (amino acids 68–137) containing a Tobacco Etch Virus (TEV) protease cleavage site after protein A was expressed from a codon optimized synthetic gene inserted into a pETM11-based vector (Genscript). A 10 mL overnight preculture of freshly transformed *Escherichia coli* BL21(DE3) Star competent cells was transferred to 1L standard lysogeny broth (LB) media containing kanamycin and grown to an OD_600_ of 0.6–0.8 at 37°C before induction with 1 mM isopropyl β-D-1-thiogalactopyranoside (IPTG), and further expressed for 16 h at 20°C and 160 rpm. For NMR experiments, 10 mL overnight precultures were transferred to minimal media (100 mM KH_2_PO_4_, 50 mM K_2_HPO_4_, 60 mM Na_2_HPO_4_, 14 mM K_2_SO_4_, 5 mM MgCl_2_; pH 7.2 adjusted with HCl and NaOH with 0.1 dilution of trace element solution (41 mM CaCl_2_, 22 mM FeSO_4_, 6 mM MnCl_2_, 3 mM CoCl_2_, 1 mM ZnSO_4_, 0.1 mM CuCl_2_, 0.2 mM (NH_4_)_6_Mo_7_O_24_, 17 mM EDTA)) supplemented with 1 g of ^15^NH_4_Cl (Sigma), and either with 6 g of ^12^C_6_H_12_O_6_ or 2 g of ^13^C_6_H_12_O_6_ (Cambridge Isotope Laboratories), followed by a growth as described for unlabeled protein. Cells were harvested (6,000 rpm for 10 min at 4°C), transferred to a denaturing lysis buffer (50 mM Tris-HCl pH 7.5, 150 mM NaCl, 20 mM imidazole, 6M urea), and sonicated (70% amplitude, 1 s pulse for 12 min on ice bath with Qsonica MC-18 sonicator). His_6_-protein A-tagged CIRBP-RGG was purified using nickel-nitrilotriacetic (Ni-NTA) agarose resin (Qiagen) and eluted in buffer containing 50 mM Tris-HCl pH 7.5, 1 M NaCl, 500 mM imidazole, 2 mM tris(2-carboxyethyl)phosphine (TCEP), 0.04% NaN_3_. The eluted protein was desalted to buffer 50 mM Tris-HCl pH 7.5, 150 mM NaCl, 20 mM imidazole, 2 mM TCEP, 0.04% NaN_3_, and subjected to overnight TEV treatment at 4°C. Cleaved CIRBP-RGG was loaded onto a HiTrap Heparin HP column (GE Healthcare), and eluted with a linear gradient of 0–100% high salt buffer (50 mM Tris-HCl pH 7.5, 1 M NaCl, 20 mM imidazole, 2 mM TCEP, 0.04% NaN_3_) over 10 column volumes (CVs). A final size exclusion chromatography purification step was performed in the buffer of interest on a Superdex 75 Increase 10/300 GL column (GE Healthcare) at room temperature.

Codon optimized synthetic His_6_-protein A-tagged MBP-CIRBP-EGFP gene was inserted into a pETM11-based vector containing a TEV protease cleavage site after protein A (Genscript). For expression of recombinant protein, the construct was transformed into *E. coli* BL21(DE3) Star cells, and grown in LB medium at 37°C. At an OD_600_ of 0.8, cells were induced with 1 mM IPTG and grown for 16 h at 20°C. Cells were harvested and lysed by sonication in a non-denaturing lysis buffer (50 mM Tris-HCl pH 7.5, 150 mM NaCl, 20 mM imidazole, 2 mM TCEP, 10% (v/v) glycerol). Following sonication, 0.1 mg/mL RNase A and MgCl_2_ (to a final concentration 20 mM) were added to the mixture and incubated in the dark for 30 min before centrifugation (13,000 g for 45 min at 4°C). His_6_-protein A-tagged MBP-CIRBP-EGFP was purified using Ni-NTA beads (Qiagen), and the eluted protein was desalted to buffer 50 mM Tris-HCl pH 7.5, 150 mM NaCl, 20 mM imidazole, 2 mM TCEP, 0.04% NaN_3_, and subsequently subjected to overnight TEV treatment at 4°C. Cleaved MBP-CIRBP-EGFP was then isolated by a second affinity purification using Ni-NTA beads. The eluted protein was then buffer exchanged to a phosphorylation buffer (50 mM Tris-HCl pH 6.7, 150 mM NaCl, 20 mM MgCl_2_, 2 mM TCEP, 0.04% NaN_3_) using HiPrep 26/10 Sephadex G-25 desalting column (GE Healthcare).

Recombinant His_6_-protein A-tagged SRPK1 containing a TEV protease cleavage site after protein A was expressed from a codon optimized synthetic gene inserted into a pETM11-based vector (Genscript). 10 mL of overnight precultures of freshly transformed *E. coli* BL21(DE3) Star cells were added to and grown in 1L LB media at 37°C until an OD_600_ reached ∼0.6–0.8, and the expression was induced with 1 mM IPTG for 16 h at 20°C. Cells were harvested at 6,000 rpm for 10 min at 4°C, and lysed by sonication in the non-denaturing lysis buffer. His_6_-protein A-tagged SRPK1 was applied on Ni-NTA beads (Qiagen), eluted to buffer 50 mM Tris-HCl pH 7.5, 1 M NaCl, 500 mM imidazole, 2 mM TCEP, 0.04% NaN_3_, desalted to buffer 50 mM Tris-HCl pH 7.5, 150 mM NaCl, 20 mM imidazole, 2 mM TCEP, 0.04% NaN_3_ at 4°C, and subjected to overnight TEV treatment at 4°C. Cleaved SRPK1 was applied on a Superdex 200 Increase 10/300 GL (GE Healthcare) size exclusion chromatography column and eluted into the phosphorylation buffer. Fractions corresponding to untagged SRPK1 were identified by SDS PAGE gel, and used immediately for experiments.

Recombinant rat His_6_-PRMT1 (amino acids 11–353) was inserted into a pET28b-His_6_ vector (Novagen) and the expression has been previously described in (Zhang and Cheng, 2003). The expression construct was transformed into *E. coli* BL21(DE3) Star cells, and 1L expression culture was grown in LB medium at 37°C. Cells were induced at an OD_600_ of 0.6–0.8 with 1 mM IPTG followed by protein expression for 16 h at 20°C. Cell pellets were harvested and sonicated in the non-denaturing lysis buffer. His_6_PRMT1 was purified using 5 mL HisTrap HP column (GE Healthcare) at 4°C and eluted over 10 CVs into buffer containing 50 mM Tris-HCl pH 7.5, 1 M NaCl, 500 mM imidazole, 2 mM TCEP, 0.04% NaN_3_. As a final polishing step size exclusion chromatography purification step was performed in a methylation buffer (50 mM Na_2_HPO_4_/NaH_2_PO_4_ pH 8.0, 150 mM NaCl, 2 mM dithiothreitol (DTT), 0.04% NaN_3_) using Superdex 200 Increase 10/300 GL column (GE Healthcare) at 4°C. Fractions corresponding to PRMT1 were identified by SDS PAGE gel, and used immediately for experiments.

For expression of recombinant unlabeled His_6_-protein A-tagged TNPO1 containing a TEV protease cleavage site after protein A, a codon optimized synthetic gene was inserted into a pETM11-based vector (Genscript). *E.coli* BL21(DE3) Star strain cells were transformed with the expression vector, and picked one colony was grown in 20 mL LB medium for 16 h at 37°C. 1 mL of pre-culture was grown for 3 days in 1L minimal medium supplemented with 6 g of ^12^C_6_H_12_O_6_ (Cambridge Isotope Laboratories) and 3 g of ^14^NH_4_Cl (Sigma) at 30°C. Cells were diluted to an OD_600_ of 0.8 and induced with 0.5 mM IPTG followed by TNPO1 expression for 6 h at 30°C. Cells pellets corresponding to protein expression of the unlabeled folded protein TNPO1 were harvested and sonicated in the non-denaturing lysis buffer. ZZ-His_6_ TNPO1 were then purified using Ni-NTA agarose beads (Qiagen) in 50 mM Tris pH 7.5, 150 mM NaCl, 20 mM imidazole, 2 mM TCEP. The eluted ZZ-His_6_ TNPO1 was subjected to TEV protease cleavage overnight at 4°C. TEV-cleaved recombinant protein was separated from the His_6_-tag using a second step of Ni-NTA purification. A final size exclusion chromatography purification step was performed in buffer containing 50 mM Tris·HCl pH 7.5, 150 mM NaCl, 2 mM TCEP, 0.04% NaN_3_ on a Hiload 16/600 Superdex 200 pg (GE Healthcare) column.

For expression of recombinant His_6_-TEV protease, *E. coli* BL21(DE3) Star cells were transformed with the pLIC-His_6_ expression plasmid (Cabrita et al., 2007) and grown in standard LB medium. Protein expression was induced at OD_600_ of 0.8 with 1 mM IPTG and left overnight at 20°C to grow. Cells were lysed in TEV lysis buffer (50 mM Tris pH 8.0, 200 mM NaCl, 25 mM imidazole, 10% (v/v) glycerol, supplemented 30 min prior sonication with 2 mM MgCl_2_, 2 µl benzonase, and 50 µl bacterial protease cocktail inhibitor added per 1L culture) by sonification. Next, His_6_-TEV was purified using Ni-NTA beads, washed using TEV lysis buffer containing 1.0 M NaCl, and eluted in TEV lysis buffer (pH 8.5) containing 800 mM imidazole. His_6_-TEV was subsequently buffer exchanged using HiPrep 26/10 desalting column (GE Healthcare) against storage buffer (50 mM Tris pH 7.5, 150 mM NaCl, 20% glycerol, 2 mM DTT), and the protein was stored at −80°C until further use.

The concentration of proteins was estimated from their absorbance at 280 nm, using the molar extinction coefficient ε at 280 nm predicted by ProtParam tool (Gasteiger et al., 2005), assuming that the ε at 280 nm was equal to the theoretical ε value.

### HEK293T Cell Lysate Phosphorylation

HEK293T cells were grown in Dulbecco’s modified Eagle’s medium (DMEM) (Sigma-Aldrich) containing 10% fetal bovine serum (FBS) (Gibco; Thermo Fisher Scientific), penicillin (100 U/mL, Gibco), streptomycin (100 µg/mL, Gibco), and amphotericin B (1.25 µg/mL; Gibco) in a humidified incubator (37°C, 5% CO_2_/95% air). HEK293T cells were lysed in 50 mM Tris-HCl pH 7.5, 150 mM NaCl, 2 mM TCEP, 1% (v/v) Triton by incubating for 30 min at 4°C with vortexing every 5 min. The HEK293T cell lysate was then centrifuged at 13,000 rpm for 30 min at 4°C, and total protein concentration was estimated using Bradford protein assay (Bradford, 1976). To perform phosphorylation reaction, ^13^C-^15^N-labeled 50 µM His_6_-protein A-tagged CIRBP-RGG was incubated overnight at room temperature with 15 mg/mL of total protein obtained from HEK293T-whole-cell-lysate in the presence of a protease inhibitor (Roche), phosphatases inhibitor (Roche), 10 mM ATP, and 10 mM MgCl_2_. On the following day, the His_6_-protein A-tagged CIRBP-RGG sample was repurified by applying on Ni-NTA agarose beads (Qiagen) and eluted in 50 mM Tris-HCl pH 7.5, 1.0 M NaCl, 500 mM imidazole, 2 mM TCEP, 0.04% NaN_3_. The eluted protein was subjected to overnight TEV treatment at 4°C, and on the next day cleaved CIRBP-RGG was desalted to 50 mM Tris-HCl pH 7.5, 150 mM NaCl, 20 mM imidazole, 2 mM TCEP, 0.04% NaN_3_ and isolated by a second affinity purification using Ni-NTA beads. As a final polishing step size exclusion chromatography purification step was performed in 50 mM Tris-HCl pH 6.7, 150 mM NaCl, 2 mM TCEP, 0.04% NaN_3_ (Superdex 75 Increase 10/300 GL, GE Healthcare) at room temperature.

### *In vitro* Phosphorylation

Recombinant CIRBP-RGG, CIRBP-EGFP and SRPK1 were equilibrated in the phosphorylation buffer. CIRBP-RGG and CIRBP-EGFP were *in vitro* phosphorylated by incubating overnight at room temperature with SRPK1 and 10 mM adenosine triphosphate (ATP), added just prior the reaction start. SRPK1 was used at a molar ratio of 1:2 for CIRBP-RGG and CIRBP-EGFP, and phosphorylation reaction was analyzed using ^1^H-^15^N HSQC spectra. Phosphorylated CIRBP-RGG (pCIRBP-RGG) was then isolated from SRPK1 by heating the sample at 95°C for 10 min and performing a size exclusion chromatography in the buffer of interest (Superdex 75 Increase 10/300 GL, GE Healthcare).

### *In vitro* Methylation

The respective gel filtration fractions of CIRBP-RGG and PRMT1 eluted into the methylation buffer were collected and used for *in vitro* methylation. CIRBP-RGG was *in vitro* methylated by incubating with PRMT1 and 2 mM S-adenosyl-L-methionine (SAM) overnight at room temperature. PRMT1 was used at a molar ratio of 1:2 for CIRBP-RGG, and the methylation reaction was analyzed by NMR ^1^H-^13^C HSQC spectra. To remove PRMT1, methylated CIRBP-RGG (metCIRBP-RGG) sample was heated for 10 min at 95°C and applied on size exclusion chromatography column in the buffer of interest (Superdex 75 Increase 10/300 GL, GE Healthcare).

### Stress Granule Association Assay in Semi-Permeabilized Cells

The SGs association assay was performed as described in Hutten and Dormann (2020). HeLa cells were maintained in DMEM high glucose GlutaMAX (Invitrogen) supplemented with 10% FBS and 50 µg/mL gentamicin at 37°C, 5% CO_2_ in a humified incubator. For the SG association assay, cells were grown on high precision, poly-L-lysine (Sigma) coated 12 mm coverslips and SGs induced by 10 µM MG132 treatment for 3h. Cells were then permeabilized 2 times 2 min each with 0.004–0.005% digitonin (Calbiochem) in KPB (20 mM potassium phosphate pH 7.4, 5 mM Mg(OAc)_2_, 200 mM KOAc, 1 mM EGTA, 2 mM DTT and 1 mg/mL each aprotinin (Roth), pepstatin (Roth) and leupeptin (Roth)). After several washes to remove soluble proteins (4 times 4 min in KPB on ice), nuclear pores were blocked by 15 min incubation with 200 µg/mL wheat germ agglutinin (WGA) on ice. Cells were then incubated for 30 min at room temperature with 200 nM CIRBP-EGFP (non- vs phosphorylated and unmethylated vs arg-methylated, respectively) diluted in KPB buffer. For SG association of phosphorylated CIRBP, protein samples were normalized for concentration of ATP and thus differed only in the presence or absence of SRPK (final conc: 100 nM). Note that unmethylated CIRBP contained the same amount of PRMT1 as methylated CIRBP. Subsequently, cells were washed (3 times 5 min in KPB on ice) to remove unbound CIRBP-EGFP. SGs were subsequently subjected to immunofluorescence for G3BP1 as a marker of SGs. For this, cells were fixed in 3.7% formaldehyde/PBS buffer for 7 min at RT and permeabilized in 0.5% TX-100/PBS for 5 min at room temperature. Cells were blocked for 10 min in blocking buffer (1% donkey serum in PBS/0.1% Tween-20) and incubated with primary antibody (rabbit anti-G3BP1, Proteintech, cat.no.13057-2-AP) in blocking buffer for 45–60 min at RT. Secondary antibodies (Alexa 555 Donkey-anti-Rabbit; Thermo, cat.no. A-31572) were diluted in blocking buffer and incubated for 30 min at room temperature. Washing steps after antibody incubation were performed with PBS/0.1% Tween-20. DNA was stained with DAPI (Sigma) at 0.5 mg/mL in PBS and cells mounted in ProLong Diamond Antifade (Thermo). Cells were imaged by confocal microscopy using identical settings for reactions within the same experiment (Performed as described in Hutten and Dormann (2020)).

### Stress Granule Enrichment in Intact Cells

For generation of the CIRBP 3D and 3A constructs, synthetic gBlocks (IDT) harboring either S-to-D or S-to-A mutations at the positions Ser97, Ser115 and Ser130 were cloned into the KpnI and BamHI sites of the GCR_2_-GFP_2_-CIRBP wt construct (Bourgeois et al., 2020). HeLa cells were grown for at least two passages in DMEM supplemented with 10% dialyzed FCS (Thermo) and transiently transfected with GCR_2_-GFP_2_-CIRBP wt, 3D or 3A constructs using Lipofectamine 2000 (Thermo). One day after transfection, cytoplasmic condensates formed likely either as a response to transfection stress or by CIRBP overexpression were stained by G3BP1 immunostaining, and enrichment of the GCR_2_-GFP_2_-CIRBP reporter in those cytoplasmic condensates was analyzed.

### Confocal Microscopy

For SG association of phosphorylated CIRBP in semi-permeabilized cells, confocal microscopy was performed at the Bioimaging core facility of the Biomedical Center, LMU Munich with an inverted Leica SP8 microscope, equipped with lasers for 405, 488, 552 and 638 nm excitation. Images were acquired using two-fold frame averaging with a 63x1.4 oil objective, and an image pixel size of 59 nm. The following fluorescence settings were used for detection: DAPI: 419–442 nm, GFP: 498–563 nm, Alexa 555: 562–598 nm. Recording was performed sequentially to avoid bleed-through using a conventional photomultiplier tube. For SG association of methylated CIRBP in semi-permeabilized cells and of phosphomutants of CIRBP in intact cells, confocal microscopy was performed at the Light Microscopy Core Facility of the Biocenter at JGU Mainz with an inverted Leica SP5 microscope using lasers for 405 nm, 488 nm (Argon line) and 561 nm for excitation. Images were acquired with bidirectional scanning using two-fold frame averaging with an 100x/1.3 Oil objective and an image pixel size of 60.6 nm. The following fluorescence settings were used for detection: DAPI: 419–442 nm, GFP: 498–563 nm, Alexa 555: 571–598 nm. Recording was performed using a conventional photomultiplier tube for DAPI and Alexa 555 and a Hybrid Detector (HyD) for GFP signals.

### Quantification of CIRBP-EGFP in Stress Granules

For quantitative measurements, equal exposure times and processing conditions for respective channels were applied to all samples within one experiment, and acquired images were quantified using ImageJ/Fiji. For quantification of CIRBP SG association in semi-permeabilized cells (performed as described in Hutten and Dormann (2020)), ROIs corresponding to SGs were identified using the wand tool by G3BP1 staining and mean fluorescence intensity in the EGFP channel was determined. For each condition, at least 10 cells and at least 44 SGs were analyzed. To determine the enrichment of CIRBP wt and phosphomutants in intact cells, the ROI corresponding to ∼at least 200 G3BP1-positive cytoplasmic condensates was determined by G3BP1 staining as described above, while a band of 0.98 pixels around the condensate was used as a representative area for the cytoplasm. Fluorescence intensity values obtained for the band around the condensate (cytoplasmic intensity) were used as a proxy for expression levels. All values were background corrected and statistical analyses were performed in GraphPad Prism 8.

### NMR Spectroscopy

All NMR experiments were conducted at 25°C on Bruker 600- and 700-MHz spectrometers equipped with TXI or a TCI triple-resonance cryoprobe using between 50 and 500 µM of ^1^H-^15^N or ^1^H-^15^N-^13^C – labeled CIRBP-RGG. All spectra were processed using TopSpin 4.0.9. In particular, 1D ^1^H spectra were processed in Mnova 11, 2D heteronuclear spectra were analyzed with the use of NMRFAM-Sparky 3.114 (Lee et al., 2015b) and CcpNMR 3.0.3 (Skinner et al., 2016) software, and triple resonance assignment was performed using CcpNMR 2.4.2 (Vranken et al., 2005). For assignment of *in vitro* phosphorylated and methylated residues in the CIRBP-RGG, we used the previously deposited data corresponding to the ^1^H-^15^N chemical shift backbone assignment of CIRBP-RGG (Biological Magnetic Resonance Data Bank (https://www.bmrb.wisc.edu/) entry: 28027) (Bourgeois et al., 2020). In addition, we acquired the following experiments in order to identify the methylated and phosphorylated residues: ^1^H-^15^N HSQC, ^1^H-^13^C HSQC, (H)CC(CO)NH, CBCA(CO)NH, HN(CA)NNH(N), and HN(CA)NNH(H). Except *in vitro* methylation, all experiments were performed using protein samples prepared in 50 mM Tris-HCl pH 6.7, 150 mM NaCl, 2 mM TCEP, 0.04% NaN_3_ (including 20 mM MgCl_2_ for *in vitro* phosphorylation experiments), and 10% (v/v) deuterium oxide was added for the lock signal in all samples. Processing and analysis of time-resolved 2D NMR spectra was performed as described in Theillet et al. (2013), and the plotted NMR signal intensities corresponding to modified residues were normalized by the sum of respective signal intensities in the reference and final spectra.

### Turbidity Assay

CIRBP-RGG, pCIRBP-RGG and RNA (12 × UG repeats) samples were prepared in 50 mM Tris-HCl, pH 7.5, 150 mM NaCl, 2 mM TCEP, 0.04% NaN_3_. Turbidity measurements were conducted at 620 nm in 96-well plates with 90-μL samples using a BioTek Power Wave HT plate reader (BioTek).

### Differential Interference Contrast Microscopy

CIRBP-RGG, pCIRBP-RGG and RNA (12 × UG repeats) samples were prepared in 50 mM Tris·HCl, pH 7.5, 150 mM NaCl, 2 mM TCEP, 0.04% NaN_3_.The 30-μL sample was plated on a 30-mm No. 1 round glass coverslip and mounted on an Observer D1 microscope with 100×/1.45 oil immersion objective (Zeiss). Protein droplets were viewed using HAL100 halogen lamp, and images were captured with an OrcaD2 camera (Hamamatsu) using VisiView 4.0.0.13 software (Visitron Systems GmbH). Droplet formation was induced by the addition of RNA for all proteins, and pictures were recorded for 30 min after addition of RNA.

### Isothermal Titration Calorimetry

All proteins samples were equilibrated in the same buffer containing 50 mM Tris·HCl, pH 7.5, 150 mM NaCl, 5 mM TCEP, 0.04% NaN_3_. Isothermal titration calorimetry (ITC) measurements were taken with a MicroCal VP-ITC instrument (Microcal) with 28 rounds of 8-μl injections at 25°C. Integration of peaks corresponding to each injection, subtraction of the contribution of protein dilution, and correction for the baseline were performed using the Origin-based 7.0 software provided by the manufacturer. Curve fitting was done with a standard one-site model and gives the equilibrium binding constant (Ka) and enthalpy of the complex formation (ΔH).

## Results

### Serine-Arginine Protein Kinase-1 Phosphorylates Multiple Sites Within CIRBP-RGG

Arginine methylation in the RG/RGG regions of RNA-binding proteins (RBPs), such as FUS or CIRBP, has been previously shown to suppress their phase separation and stress granule (SG) recruitment, as well as to modulate binding to nuclear importins (Hofweber et al., 2018; Qamar et al., 2018). As several RBPs have been reported to be phosphorylated (Toyota et al., 2010; Nonaka et al., 2016; Monahan et al., 2017; Reineke et al., 2017), we hypothesized that phosphorylation of their low-complexity region could also regulate their LLPS and membrane-less organelles association.

**FIGURE 1:**
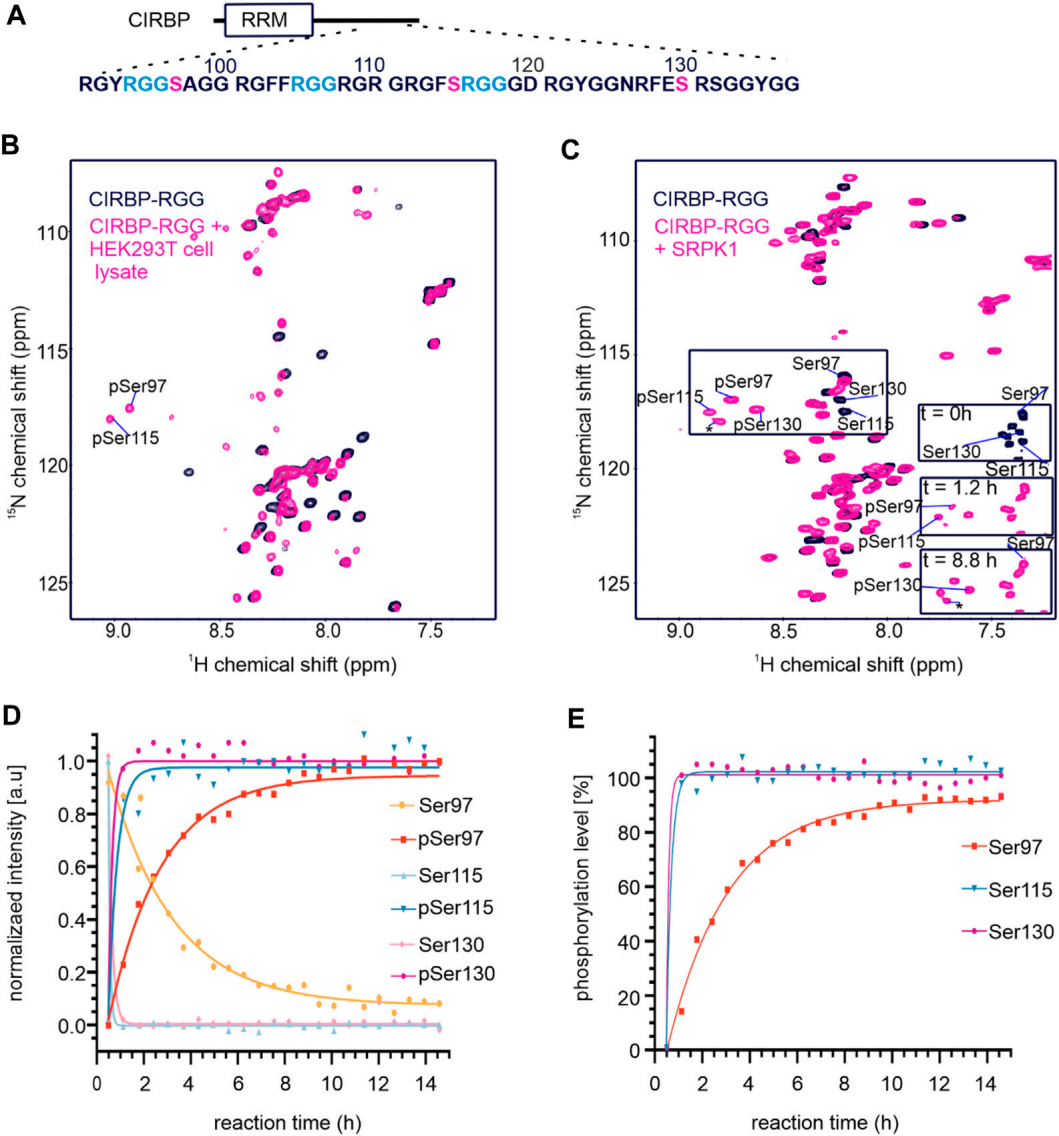
CIRBP-RGG is phosphorylated by SRPK1 *in vitro*. **(A)** Architectural organization of CIRBP showing the RRM (RNA-recognition motif) and the sequence of the CIRBP-RGG containing the three RGGs (blue) with adjacent serine residues (magenta). **(B)** Overlay of ^1^H-^15^N HSQC spectra of ^15^N-labeled CIRBP-RGG (black) with repurified ^13^C-^15^N-labeled CIRBP-RGG after incubation with HEK293T whole-cell lysate (magenta). Cross peaks for phosphorylated serine residues assigned by triple resonance experiments are labeled. **(C)** ^1^H-^15^N HSQC spectrum of 100 µM ^15^N-labelled CIRBP-RGG in the absence (black) and presence of 50 µM SRPK1 and 10 mM ATP (magenta). Resonance peaks corresponding to phosphorylated serine residues are marked. Both appearance of phosphoserines and disappearance of the corresponding serine signal are shown at the indicated time points on a bottom right part of the spectrum. The cross peak labeled with an asterisk could not be assigned by triple resonance experiments, and may correspond to either an intermediate phosphorylation state, or to non-assigned phosphosite (e.g. Ser132 is a phosphosite reported in PhosphoSitePlus database, and the shift of its resonance peak was detected in NMR experiments (not shown)). **(D)** Change of NMR cross peak signal intensity of both appearing phosphoserines and disappearing serines is shown over time (sample from Figure 1C). **(E)** The graph shows the calculated phosphorylation level for serines 97, 115, and 130 in CIRBP-RGG after incubation with SRPK1 (sample from Figure 1C).

To investigate how phosphorylation in the RG/RGG region of RBPs regulates their LLPS and membrane-less organelles association, we focused on the RG/RGG region of CIRBP (CIRBP-RGG) as it contains serine residues neighboring the low-complexity arginine/glycine-rich regions in its primary sequence (Figure 1A). These serine residues may constitute potential phosphorylation sites. NMR spectroscopy is well-suited to study PTMs providing residue-resolved and kinetic information on the post-translationally modified sites (Theillet et al., 2012). Thus, we investigated the effects of treating recombinant CIRBP-RGG with a cell lysate (containing various kinases) obtained from HEK293T cells by applying solution NMR spectroscopy (Figure 1B). As recombinant CIRBP-RGG was purified from bacterial cells, the protein was originally non-phosphorylated. ^1^H-^15^N heteronuclear single quantum coherence (HSQC) spectra show that after the incubation of the HEK293T whole-cell lysate with ^13^C-^15^N-isotopically labeled CIRBP-RGG, downfield ^1^H-^15^N resonance peaks appear. With the use of triple-resonance NMR experiments, the new peaks were assigned to phosphorylated residues Ser97 and Ser115. Both residues are located in the proximity of the CIRBP RG/RGG region (Figure 1A). These data indicate the presence of enzymatically active serine kinases in the cell lysate phosphorylating serine residues in CIRBP-RGG. We speculated that serine-arginine (SR) protein kinase-1 (SRPK1) phosphorylates CIRBP, as it is known to exhibit a robust phosphorylation activity of serine residues in serine/arginine (SR)-rich protein regions (Ghosh and Adams, 2011; Bullock and Oltean, 2017; Patel et al., 2019).

To address our hypothesis, we established an *in vitro* phosphorylation protocol where purified SRPK1 was incubated with recombinant CIRBP-RGG and adenosine triphosphate (ATP) as a phosphate donor. We examined SRPK1-mediated phosphorylation of ^15^N-labeled CIRBP-RGG by performing NMR spectroscopy, and monitored the appearance of characteristic downfield ^1^H-^15^N NMR cross peaks corresponding to phosphoserine residues (Figure 1C). These residues were assigned as pSer97, pSer115 and pSer130. By monitoring the NMR signal intensity of disappearing NMR cross peaks for Ser97, Ser115, Ser130 and appearing resonances for the phospho-residues, we observed that the fully phosphorylated state of serines 115 and 130 is reached within 1 h, whereas the plateau of the maximal NMR intensity for pSer97 is reached after approximately 5 h (Figures 1D,E). Both serine residues 115 and 130 are located within the consensus recognition motif for SRPK1 (dipeptide serine-arginine) explaining their faster phosphorylation compared to serine 97, which is separated by two glycine residues from arginine (Figure 1A). So far, phosphorylation of serine residues 97 and 115 has not been reported in databases such as iPTMnet (Huang et al., 2018), PhosphoSitePlus (Hornbeck et al., 2015), qPTM (Yu et al., 2019), or PTMcode (Minguez et al., 2013) implying the discovery of two *de novo* phosphorylation sites in CIRBP-RGG.

In summary, we show that the RG/RGG region of CIRBP can be phosphorylated by SRPK1 *in vitro*, however the manner in which this modification impacts RG/RGG properties on a molecular level remains unknown. Therefore, we subsequently sought to explore the impact of SRPK1-mediated phosphorylation of CIRBP-RGG on its phase separation and SG association.

### Phosphorylation Suppresses *in vitro* Phase Separation of CIRBP-RGG and Stress Granules Association of CIRBP in Cells

It has been previously reported that the RG/RGG region of CIRBP phase separates *in vitro* upon addition of RNA in a concentration-dependent manner, and is essential for SG recruitment in response to cellular stresses (Bourgeois et al., 2020). Furthermore, we and others have shown that asymmetric dimethylation of the RGG3 region in FUS reduces its phase separation propensity (Hofweber et al., 2018; Qamar et al., 2018). Here, we demonstrate that CIRBP-RGG is *in vitro* phosphorylated by SRPK1, but it remains unclear whether CIRBP-RGG phosphorylation could control biologically relevant properties. Therefore, we aimed at deciphering whether SRPK1-mediated *in vitro* phosphorylation of CIRBP-RGG similarly regulates its ability to phase separate and to be recruited into SGs.

**FIGURE 2:**
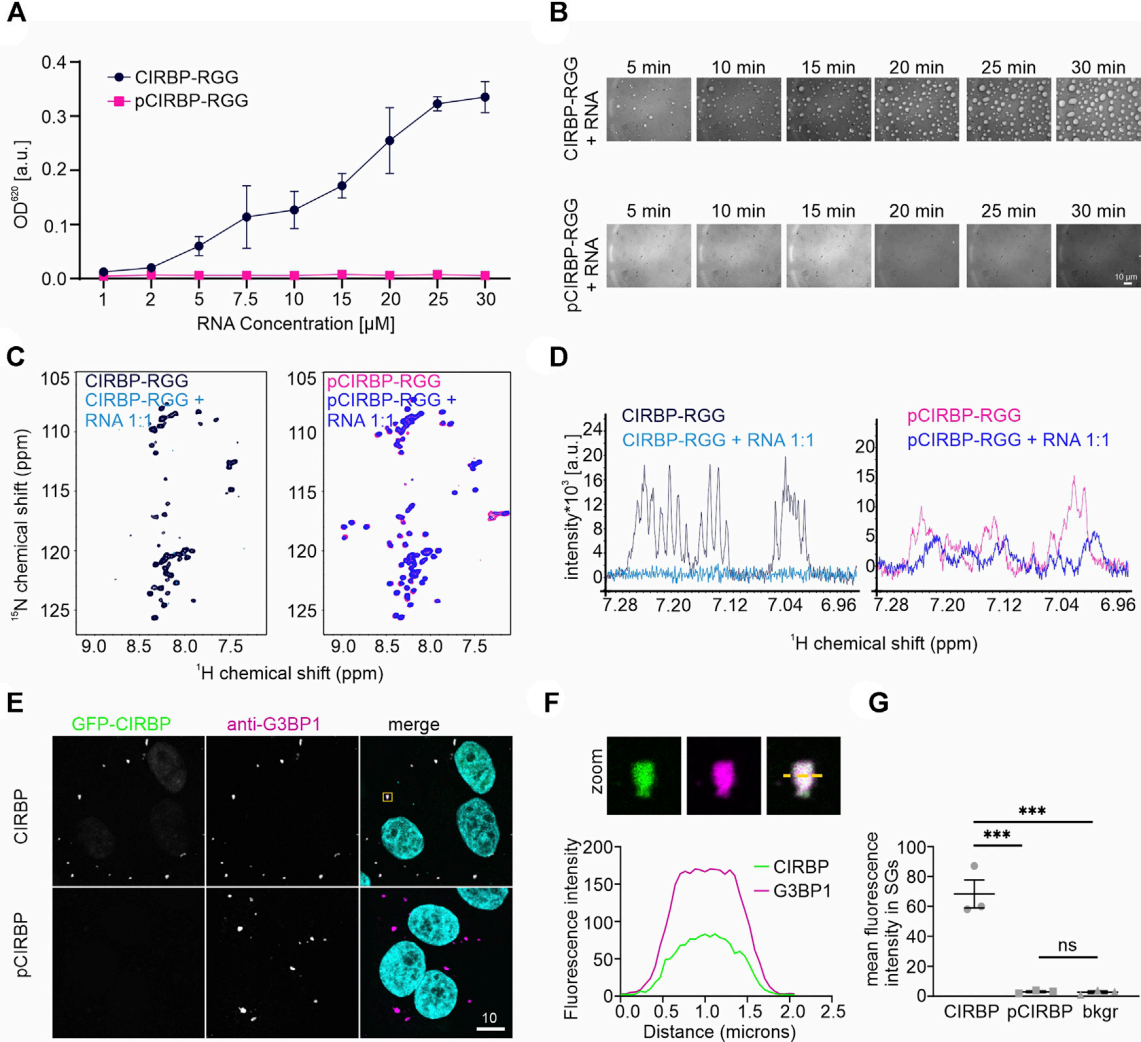
SRPK1-mediated phosphorylation of CIRBP-RGG impairs its phase separation and SGs recruitment. **(A)** Turbidity assay performed at a fixed concentration of CIRBP-RGG and pCIRBP-RGG (both at 30 µM) with an increasing concentration of (UG)_12_ RNA. **(B)** Differential interference contrast microscopy images illustrating CIRBP-RGG **(upper panel)** and pCIRBP-RGG **(bottom panel)** at a concentration 30 µM in the presence of 15 µM (UG)_12_ RNA. Images were recorded over 30 min, scale bar is 10 µm. **(C)** ^1^H-^15^N HSQC spectra of 50 µM ^15^N-labeled CIRBP-RGG **(left panel, in black)** and 50 µM ^15^N-labeled pCIRBP-RGG **(right panel, in magenta)** in the absence and presence of 50 µM (UG)_12_ RNA (in blue and dark-blue for CIRBP-RGG and pCIRBP-RGG, respectively). **(D)** ^1^H-NMR spectra of 50 µM ^15^N-labeled CIRBP-RGG **(left panel, in black)** and 50 µM ^15^N-labeled pCIRBP-RGG **(right panel, in magenta)** in the absence and presence of (UG)_12_ RNA at a 1:1 stoichiometric ratio (in blue and dark-blue for CIRBP-RGG and pCIRBP-RGG samples, respectively). The spectra were recorded immediately before the corresponding ^1^H-^15^N HSQC spectra in Figure 2C. **(E)** Association of CIRBP-EGFP **(upper panel)** and phosphorylated CIRBP-EGFP **(lower panel)** with SGs in semi-permeabilized HeLa cells. Scale bar:10 μm. Yellow box indicates the zoomed-in images shown in Figure 2F. **(F)** Unmodified CIRBP completely enters the SG as shown by a zoomed-in image of an exemplary SG and plot profiles of fluorescence intensities for G3BP1 and GFP-CIRBP along the yellow line. **(G)** Quantification of the mean fluorescence intensity of CIRBP-EGFP and phosphorylated CIRBP-EGFP in SGs for three independent replicates with ≥44 SGs ± SEM. ***p < 0.0002 by an one-way ANOVA with Tukey’s multiple comparison test.

In a turbidity assay measuring the optical density (OD) of protein solution at 620 nm, we observed that titration of increasing amounts of (UG)_12_ RNA to a fixed concentration of pCIRBP-RGG yielded no increase in turbidity (Figure 2A). In contrast and as expected, the turbidity of CIRBP-RGG in solution increases with rising amounts of added RNA. In line with our turbidity data, differential interference contrast (DIC) microscopy shows the formation of small liquid-like condensates of CIRBP-RGG in the presence of (UG)_12_ RNA, whereas the ability to phase separate was reduced in pCIRBP-RGG (Figure 2B). This demonstrates the inhibitory role of *in vitro* phosphorylation on CIRBP-RGG phase separation. To confirm the aforementioned findings, we examined the effects of (UG)_12_ RNA incorporation to a ^15^N-labeled CIRBP-RGG or pCIRBP-RGG in solution by means of NMR spectroscopy. Addition of 1.0 stoichiometric equivalent of RNA caused a substantial decrease of CIRBP-RGG NMR cross peak signal intensity (Figure 2C). This is in line with previous data reporting the formation of high-molecular weight RG/RGG:RNA droplets (Bourgeois et al., 2020). Interestingly, a decrease of NMR signal intensity in the corresponding one-dimensional ^1^H-NMR spectra after the addition of (UG)_12_ RNA to CIRBP-RGG and pCIRBP-RGG is also observed, suggesting that although pCIRBP-RGG has a reduced propensity to phase separate *in vitro* it still can bind to RNA (Figure 2D).

**FIGURE 3:**
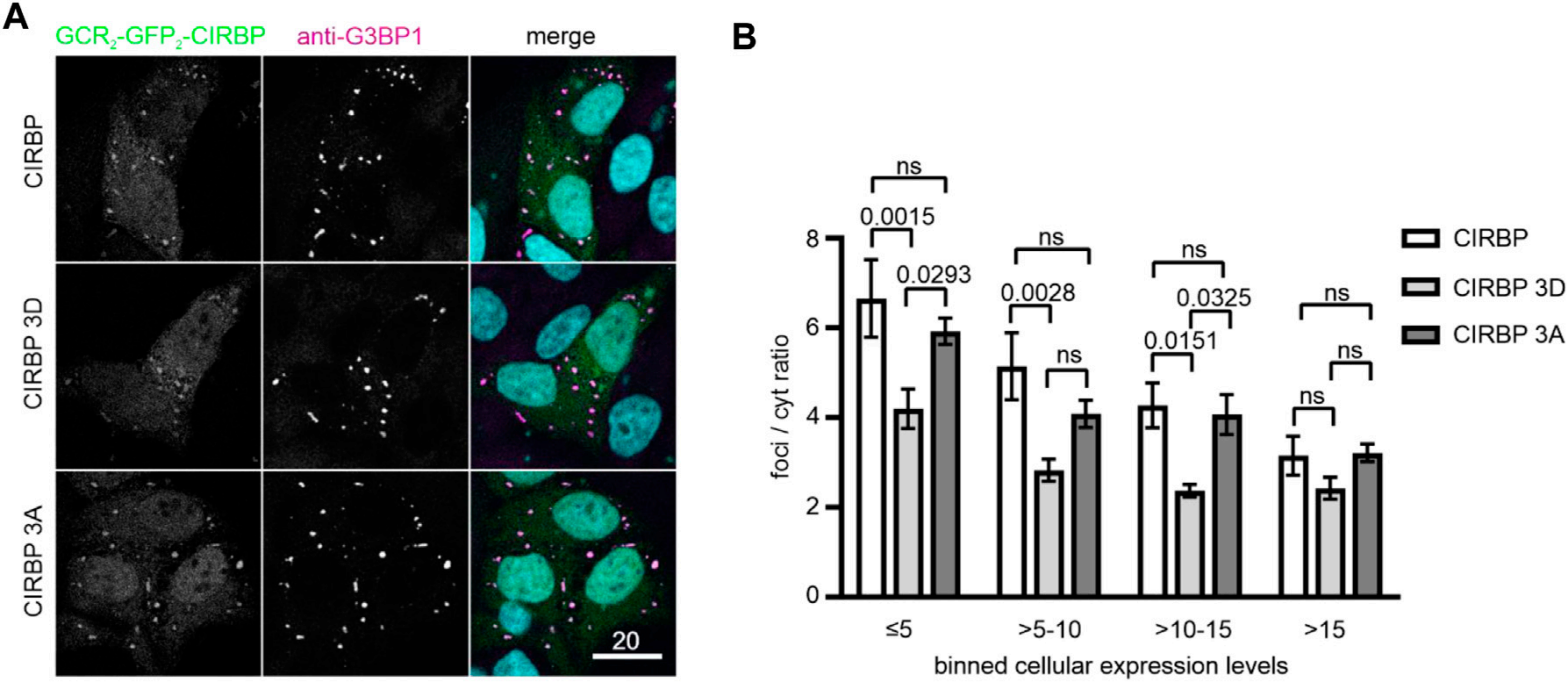
Recruitment of CIRBP into G3BP1-positive condensates in intact cells depends on phosphorylation of the RG/RGG region. **(A)** Association of GCR_2_-GFP_2_-CIRBP **(upper panel),** GCR_2_-GFP_2_-CIRBP 3D mutant **(middle panel),** and GCR_2_-GFP_2_-CIRBP 3A mutant **(bottom panel)** with cytoplasmic condensates positive for G3BP1 in HeLa cells. Scale bar: 20 μm. **(B)** Quantification of enrichment of GCR_2_-GFP_2_-CIRBP wt, 3D or 3A mutant in G3BP1-positive condensates over the cytoplasm (foci/cyt ratio) as a mean of 4 independent replicates ± SEM depending on the cellular expression levels represented in bins of fluorescence intensity units, adjusted *p*-values by 2-way ANOVA with Tukey’s multiple comparisons test; ns, non-significant.

To further confirm our findings in the cellular context, we conducted a SG recruitment assay in cells semi-permeabilized by digitonin (Hutten and Dormann, 2020). We have previously reported that after adding recombinantly purified GFP- and maltose-binding protein (MBP)-tagged full-length CIRBP to semi-permeabilized cells, CIRBP accumulates in G3BP1-positive SGs (Bourgeois et al., 2020). Here, after addition of *in vitro* phosphorylated recombinant CIRBP-EGFP to semi-permeabilized cells, we observed that SG association is significantly reduced compared to the non-phosphorylated protein (Figures 2E,G). To analyze localization of CIRBP to cellular, cytoplasmic condensates in dependence of RG/RGG-region phosphorylation in intact cells, we made use of our previously described cytoplasmically anchored CIRBP reporter (GCR_2_-GFP_2_-CIRBP, (Bourgeois et al., 2020)). In this reporter, CIRBP localizes mainly in the cytoplasm due to fusion with the hormone-binding domain of the glucocorticoid receptor (GCR). When we compared enrichment of a phosphomimetic mutant form of CIRBP, in which Ser 97, 115 and 130 were replaced by aspartate; (CIRBP 3D) with CIRBP wt, we noticed a mild, but significant reduction of the enrichment for the 3D mutant to cytoplasmic condensates that stained positive for the SG protein G3BP1 (Figure 3A). Importantly, mutation of the same serines to alanines (CIRBP 3A) did not significantly affect this recruitment compared to the wildtype. While the mean expression levels of the reporters were relatively similar, we noted however, that in some replicates the 3D mutant exhibited a slightly reduced expression level compared to CIRBP wt and 3A, which could also influence the level of SG localization to some extent. Therefore, we binned data with similar expression levels to allow for a direct comparison of cells with comparable expression levels (Figure 3B) and confirmed a significant reduction of the enrichment for the 3D mutant to cytoplasmic condensates. These findings suggest that phosphorylation of the RG/RGG region also lessens recruitment of CIRBP to membraneless organelles in intact cells. We cannot exclude that other potential SRPK1 phosphorylation sites contribute to the observed SGs association impairment in the context of full-length CIRBP in our semi-permeabilized cell assay, yet our previous data demonstrated that the RG/RGG region of CIRBP, and not its C-terminal RSY regions, drives SGs association in cells (Bourgeois et al., 2020).

**FIGURE 4:**
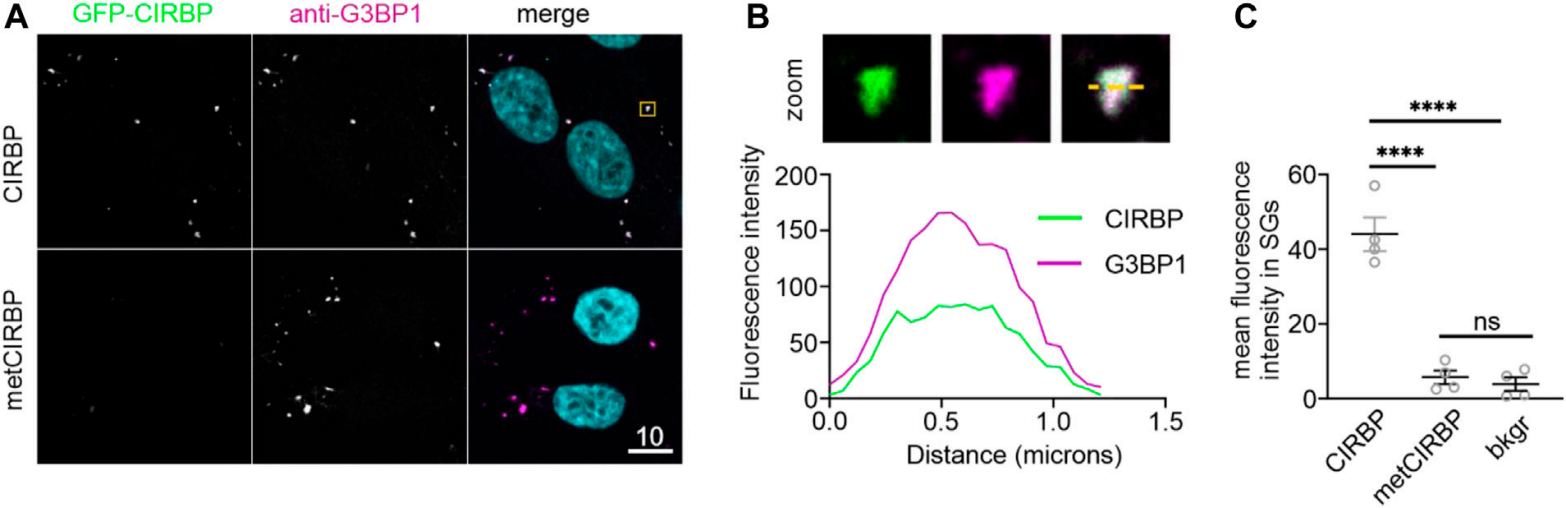
PRMT1-mediated arginine methylation of CIRBP-RGG reduces its SGs recruitment. **(A)** Association of CIRBP-EGFP **(upper panel)** and methylated CIRBP-EGFP **(metCIRBP; lower panel)** with SGs in semi-permeabilized HeLa cells. Scale bar: 10 μm. Yellow box indicates the zoomed-in images shown in Figure 4B. **(B)** Unmodified CIRBP completely enters the SG as shown by a zoomed-in image of an exemplary SG and plot profiles of fluorescence intensities for G3BP1 and GFP-CIRBP along the yellow line. **(C)** Quantification of the mean fluorescence intensity of CIRBP-EGFP and methylated CIRBP-EGFP in SGs for 4 independent experiments using CIRBP from 2 independent methylation reactions with ≥44 SGs ± SEM, adjusted *p*-value: ****p < 0.0001 by one-way ANOVA with Tukey’s multiple comparisons test; ns, non-significant.

Furthermore, considering our results demonstrating the inhibitory effects of serine phosphorylation of CIRBP-RGG on its phase separation and SGs recruitment, we proceeded to investigate how PRMT1-mediated arginine methylation of CIRBP affects its SGs association. We observe that SG recruitment of *in vitro* methylated CIRBP-EGFP in semi-permeabilized cells is substantially reduced compared to the non-methylated protein (Figure 4). Hence, our data remain in agreement with a previous study showing reduction of LLPS and SGs recruitment of methylated FUS, another RG/RGG-region containing protein (Hofweber et al., 2018), and imply that both serine phosphorylation and arginine methylation of CIRBP-RGG weaken its ability to associate with SGs.

Collectively, our data reveal that SRPK1-mediated serine phosphorylation of CIRBP-RGG reduces RNA-driven phase separation *in vitro* and suppresses SGs recruitment of CIRBP. Lastly, we uncover that arginine methylation, similarly to serine phosphorylation, reduces SG recruitment of CIRBP-EGFP, hence the biological implications of the co-existence of these two PTMs and their mutual modulation in CIRBP and other RG/RGG-region containing proteins remains yet to be discovered.

### SRPK1-mediated Phosphorylation of CIRBP-RGG Impairs its Binding to the Nuclear Import Receptor Transportin-1

The nuclear import receptor Transportin-1 (TNPO1) binds its cargoes through a proline tyrosine (PY)-NLS and an RG/RGG region to mediate nuclear import (Lee et al., 2006; Dormann et al., 2010; Bourgeois et al., 2020). We and others have shown that TNPO1 binding to RG/RGG proteins, such as FUS or CIRBP, can reduce their phase separation *in vitro* and SGs recruitment in cells, thus exerting a chaperone-like function (Guo et al., 2018; Hofweber et al., 2018; Qamar et al., 2018; Yoshizawa et al., 2018). Furthermore, arginine methylation of the RG/RGG region in CIRBP weakens its interaction with TNPO1 (Hofweber et al., 2018), but it is still unknown whether and how phosphorylation of CIRBP-RGG affects transportin-1 binding.

To address this question, we utilized isothermal titration calorimetry (ITC) to characterize binding between TNPO1 and pCIRBP-RGG. ITC analysis revealed that *in vitro* phosphorylation of CIRBP-RGG precluded the binding of TNPO1, whereas non-phosphorylated CIRBP-RGG bound TNPO1 with an ITC-derived dissociation constant (Kd) of 124.4 ± 14.8 nM (Supplementary Figures S1A,B). These results demonstrate that phosphorylation of CIRBP-RGG substantially reduces binding to TNPO1.

### RS/SR Phosphorylation Sites are Found Next to RG/RGG Regions in a Variety of Human Proteins

**FIGURE 5:**
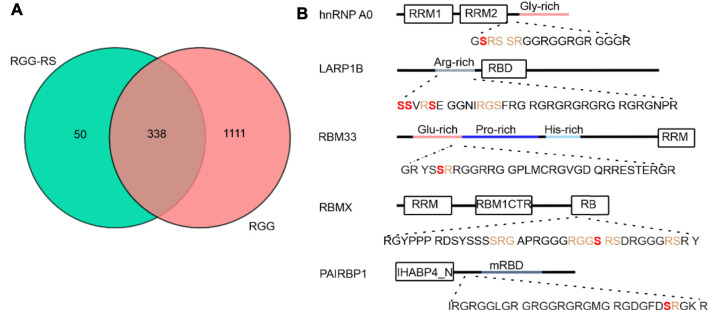
Analysis of distribution of coexisting RG/RGG and RS/SR regions in human proteome. **(A)** Venn diagram corresponding to the PROSITE analysis (https://prosite.expasy.org/scanprosite/) of two motifs from protein sequences database filtered for human proteins (taxid:9606) harboring either 1) a di-RG motif (pink) each spaced by zero to five amino acids [R-G-x(0-5)-R-G] or 2) a di-RG motif in the presence of RS or SR in the middle, after or before di-RG motif(blue) spaced by zero to five residues [R-G-x(0,5)-R-G-x(0,5)-R/S], [R/S-x(0,5)-R-G-x(0,5)-R-G] and [R-G-x(0,5)-R/S-x(0,5)-R-G]. These two groups of proteins were compared with each other in the Venn diagram. **(B)** Domain organization of five putative human proteins possessing RS/SR motifs within or next to the RG/RGG region; architectural representation was performed according to InterPro (https://www.ebi.ac.uk/interpro/protein/) and Uniprot (https://www.uniprot.org/) databases. RS/SR motifs located in the proximity to RG/RGG regions are shown in orange, and serine residues that are reported to carry phosphorylation (according to iPTM and PhosphoSitePlus) are illustrated in bold (red). Abbreviations used: hnRNP A0 - heterogeneous nuclear ribonucleoprotein A0; RRM – RNA recognition motif; LARP1B- La ribonucleoprotein domain family member 1B; RBD - HTH La-type RNA-binding domain; RBM33 - RNA-binding protein 33 (drawn not in a scale); RBMX - RNA-binding motif protein, X chromosome; RBM1CTR - C-terminal region present in RBM1-like RNA binding hnRNPs; RB- region necessary for RNA-binding; PAIRBP1 - plasminogen activator inhibitor 1 RNA-binding protein; IHABP4_N - Intracellular hyaluronan-binding protein 4, N-terminal domain; mRBD - Hyaluronan/mRNA binding family domain.

Given that the primary sequence of CIRBP-RGG contains serine residues located in the proximity to the RG/RGG region with arginine residues serving as methylation sites (Figure 1A)*,* we performed a bioinformatic analysis to address the question of how commonly RS/SR phosphorylation sites can be found within or next to RG/RGG regions in the human proteome. We discovered that 338 out of 1449 proteins containing a di-RG motif possess RS/SR sites located within a distance of 5 residues (Figure 5A). Subsequently, we examined whether serine residues that are situated within or near RG/RGG regions can be phosphorylated, and we uncovered that these serines can be modified in a similar manner as in CIRBP-RGG (Supplementary Datasets S1, S2). Of interest, we discovered that a number of the identified proteins can carry both arginine methylation and serine phosphorylation sites in their adjacent RG/RGG and RS/SR regions (examples given in Supplementary Dataset S2; Figure 5B). Taken together, our findings indicate the co-occurrence of RG/RGG and RS/SR regions in a variety of human proteins and the possible crosstalk between phosphorylation and arginine methylation within these regions. We next sought to investigate the interplay between serine phosphorylation and arginine methylation in CIRBP-RGG.

### Arginine Methylation of CIRBP-RGG Inhibits its SRPK1-mediated Phosphorylation and *vice versa*

**FIGURE 6:**
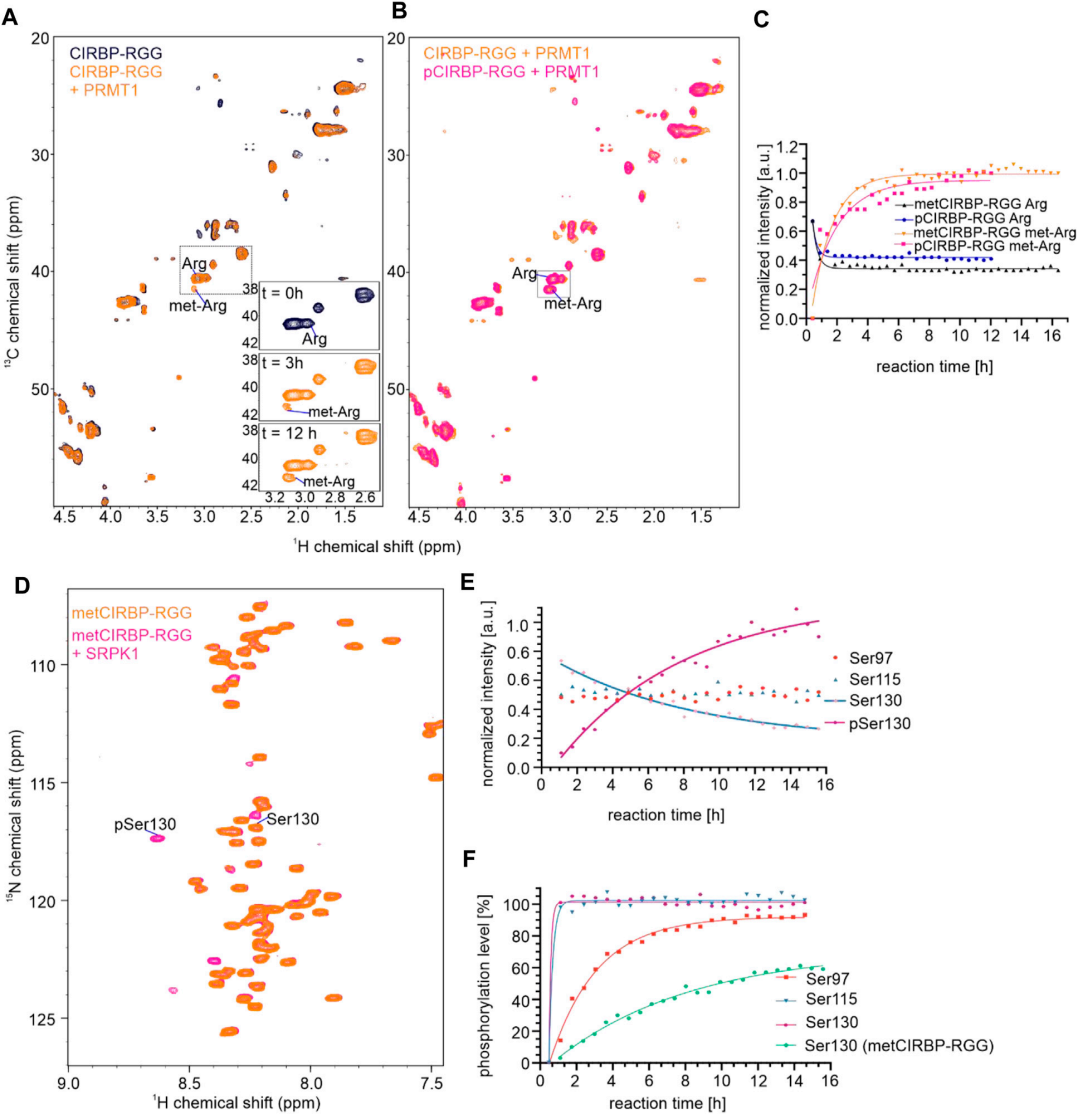
Arginine methylation of CIRBP-RGG modulates its SRPK1-mediated phosphorylation and *vice versa.* **(A)** Overlay of ^1^H-^13^C HSQC spectra of 50 µM ^13^C-^15^N-labeled CIRBP-RGG in the absence (black) and presence of 10 µM PRMT1 and 2 mM SAM (orange). The region containing a peak corresponding to methylated arginines is indicated by a dotted box, and the rising intensity of methylated arginine cross peak can be followed at the three exemplary time points shown in a bottom right part of the spectrum. **(B)** Overlay of ^1^H-^13^C HSQC spectra of ^13^C-^15^N-labeled 50 µM metCIRBP-RGG (sample as in Figure 6A; in orange) and 100 µM pCIRBP-RGG in the presence of 40 µM PRMT1 and 2 mM SAM (in magenta). The region containing a peak corresponding to methylated arginines is indicated by a dotted box. **(C)** Change of NMR signal intensities of cross peaks corresponding to methylated and non-methylated arginines in metCIRBP-RGG (in orange and black, respectively) and pCIRBP-RGG (in magenta and dark-blue, respectively) over time (samples from Figure 6B). **(D)** ^1^H-^15^N HSQC spectrum of 50 µM ^13^C-^15^N-labeled *in vitro* methylated CIRBP-RGG in the absence (orange) and presence of 25 µM SRPK1 (magenta) **(E)** Change of NMR signal intensity corresponding to cross peaks of SRPK1-phosphorylated metCIRBP-RGG serine residues (sample from Figure 6D). **(F)** Comparison of the calculated phosphorylation level of serines 97, 115 and 130 in pCIRBP-RGG (as in Figure 1E) with the only phosphoresidue (pSer130) in metCIRBP-RGG after the incubation with SRPK1 (sample from Figure 6D).

To dissect whether phosphorylation of CIRBP-RGG regulates its arginine methylation and vice versa, we recorded a series of ^1^H-^15^N-HSQC or ^1^H-^13^C-HSQC spectra over time to follow *in vitro* phosphorylation and methylation reactions, respectively, with measurements starting immediately after reconstitution of the *in vitro* system. To analyze *in vitro* methylation of phosphorylated CIRBP-RGG (pCIRBP-RGG), recombinant ^13^C,^15^N-labeled pCIRBP-RGG was applied on a gel filtration column to remove SRPK1 and transfer the protein into methylation buffer. pCIRBP-RGG was then *in vitro* methylated by addition of protein arginine methyltransferase-1 (PRMT1) and S-adenosyl-L-methionine (SAM) as a methyl group donor. Immediately after preparing the *in vitro* methylation reaction, ^1^H-^13^C-HSQC and ^1^H-^15^N-HSQC spectra were recorded and examined for the appearance of a cross peak indicative for arginine methylation (^1^H_δ_ 3.084 ppm,^13^C_δ_ 41.554 ppm) (Figures 6A,B). The signal intensity of a ^1^H-^13^C NMR cross peak corresponding to methylated arginine residues in pCIRBP-RGG reached a plateau within approximately 9 h after the reaction start, whereas for non-phosphorylated CIRBP-RGG the plateau was achieved within 7 h (Figure 6C). Consistent with reported methylarginines in iPTM/PhosphoSitePlus, our analysis of ^1^H-^13^C-HSQC and HCC(CO)NH spectra revealed that arginine residues 94, 101, 105, 112 and 116 are methylated in non-phosphorylated CIRBP-RGG (Supplementary Figure S2). Based on triple resonance assignment of methylated pCIRBP-RGG, the presence of an attached methyl group was detected in arginine residues 101, 105, 108, and 110 (Supplementary Figure S2). Resonance peaks allowing to assess the methylation status of arginine 108 and 110 appeared only in the spectrum of methylated pCIRBP-RGG, therefore we cannot draw conclusions about their methylation status in the context of the non-phosphorylated protein. Hence, the presence of a phosphate group on serine residues 97 and 115 prevented methylation of arginines 94, 112 and 116 located in the proximity to the phosphoresidues.

Moreover, we examined how arginine methylation of CIRBP-RGG affected the capacity of SRPK1 to *in vitro* phosphorylate serine residues in CIRBP. To this end, purified ^13^C-^15^N-labeled CIRBP-RGG was *in vitro* methylated and subjected to gel filtration to remove PRMT1 (metCIRBP-RGG). After the addition of SRPK1 and ATP to the solution of metCIRBP-RGG, a ^1^H-^15^N NMR cross peak (^1^H_δ_ 8.620 ppm, ^15^N_δ_ 117.318 ppm) assigned to pSer130 was detected 1 h after the reaction start (Figures 6D,E). For pCIRBP-RGG the final phosphorylation level of pSer130 was estimated to 90%, while in the case of metCIRBP-RGG this value equaled around 60% (Figure 6F) and the signal of non-phosphorylated Ser130 could still be detected (Figures 6D,E). Resonance peaks for phosphoserines pSer97 and pSer115 were not observed in phosphorylated metCIRBP-RGG (Figure 6D), and the intensity of the peaks corresponding to the non-phosphorylated species remained constant during the experiment (Figure 6E). Compared to *in vitro* phosphorylation of non-methylated CIRBP-RGG where the maximal signal intensity of the pSer130 resonance peak was achieved within approximately 1 h after the reaction start, for metCIRBP-RGG the pSer130 signal intensity did not reach a plateau after 12 h (Figures 6E,F). As serines Ser97 and Ser115 are located in the direct vicinity of arginine residues in the RG/RGG region, we suggest that the presence of methyl groups on these arginines precludes the addition of a phosphate group to a proximal serine presumably via steric effects.

In conclusion, our data show that phosphorylation of CIRBP-RGG precludes methylation of arginine residues in direct proximity to phosphoserines. Our results also indicate that arginine methylation of CIRBP-RGG prevents SRPK1-mediated phosphorylation of serines 97 and 115, and affects kinetics of phosphorylation of serine 130, which is located more distant to the RG/RGG region.

## Discussion

**FIGURE 7:**
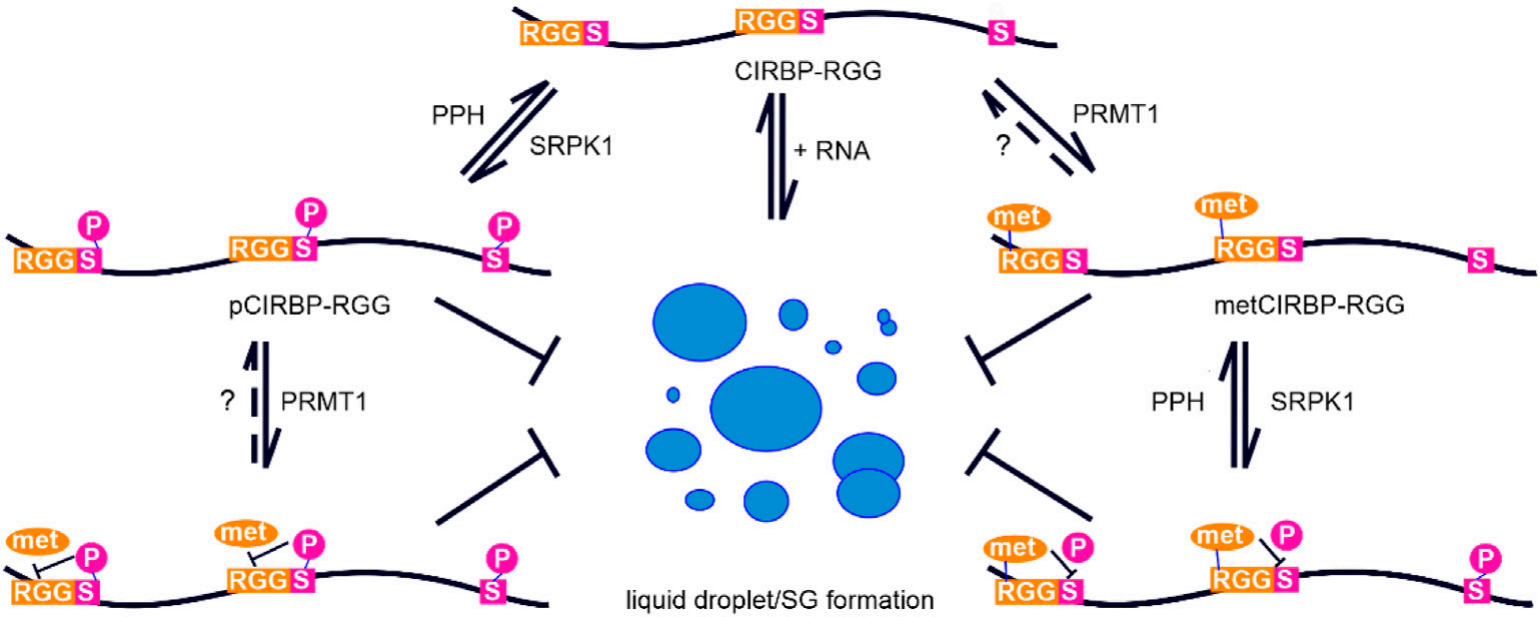
Proposed model depicting arginine methylation and phosphorylation crosstalk in CIRBP-RGG. Graph illustrating the suppression of CIRBP-RGG *in vitro* phase separation by phosphorylation and arginine methylation, as well as the crosstalk between arginine methylation and phosphorylation within CIRBP RG/RGG region. The formation of liquid droplets and biomolecular condensates is shown in a simplified manner as blue circles. Dotted line and the question mark indicate the lack of consensus regarding arginine de-methylation, and PPH represents protein phosphatases.

Here we show that CIRBP-RGG is a substrate for SRPK1-mediated phosphorylation (Figure 1). By applying NMR spectroscopy, we identified two novel phosphorylation sites in CIRBP at positions Ser97 and Ser115, where Ser97 is located outside of the consensus serine-arginine dipeptide recognition motif. Furthermore, we demonstrated that arginine methylation in the RG/RGG region of CIRBP suppresses phosphorylation of serine residues 97 and 115 by SRPK1, and the phosphorylation kinetics of phosphoserine 130 is slower compared to non-methylated CIRBP-RGG (Figures 6D–F). The presence of methyl groups on arginines might introduce a sterical hindrance that precludes SRPK1 binding and in turn inhibits phosphorylation of serines 97 and 115. We also found that SRPK1-mediated phosphorylation of CIRBP-RGG prevented methylation of arginines 94, 112 and 116 located in the proximity to phosphoserines (Figures 6B,C, Supplementary Figure S2). Aside from steric effects, the negatively charged phosphate group could interfere via electrostatic repulsion with the acidic region found in the enzymatic site of PRMTs (Zhang and Cheng, 2003). Thus, we suggest the vicinity of negatively charged phosphate groups to target arginines prevents binding to PRMT1 active site and methylation of arginines 94, 112, and 116 due to the electrostatic repulsion. This is in line with the observation that negatively charged amino acids next to the arginine disfavour methylation (Hamey et al., 2018). Hence, our findings indicate that arginine methylation and serine phosphorylation of CIRBP-RGG directly modulate each other (Figure 7).

To our knowledge, the crosstalk between arginine methylation and phosphorylation in the RG/RGG region has not been previously reported for CIRBP. It has been shown that arginine methylation within the RG/RGG region of yeast hnRNP protein Npl3p prevents phosphorylation of Npl3p by Sky1p, which is a yeast orthologue of SRPK1 (Yun and Fu, 2000; Lukasiewicz et al., 2007). Smith *et al.* recently demonstrated that Sky1p-mediated phosphorylation of the SRGG regions in *Saccharomyces cerevisiae* fibrillarin (Nop1p) blocks arginine methylation by Hmt1p (Smith et al., 2020). The authors also reported that a loss of these PTMs results in an atypical nucleolar localization. Of note, authors found that the presence of acidic residues/phosphoserine in Nop1p at positions -1, -2, (and to a smaller extent at -5 and +3) with respect to arginine in the SRGG motif negatively affects Hmt1p-mediated methylation. In contrast, we observed that phosphorylation of CIRBP-RGG did not inhibit PRMT1-methylation of Arg101 (at position +4 from pSer97) and Arg110 (at position -5 from pSer115) suggesting phosphorylation might exert more local inhibiting effects on methylation in human RG/RGG proteins. Moreover, the RG/RGG region of the herpes simplex virus 1 protein ICP27 has been demonstrated to interact with SRPK1 resulting in its translocation from the cytoplasm to the nucleus, and this interaction was decreased when arginine methylation was blocked as demonstrated by co-immunoprecipitation and co-localization studies (Souki and Sandri-Goldin, 2009). Considering the aforementioned examples of the crosstalk between arginine methylation and phosphorylation in RG/RGG proteins, by conducting a bioinformatic analysis we identified 338 di-RG motif-containing proteins that possess RS/SR sites within a five residues distance and some of them were reported to harbour simultaneously arginine methylation and phosphorylation sites. Taken together, these findings corroborate that the interplay between phosphorylation and arginine methylation in RG/RGG regions of proteins may play important roles across the RG/RGG proteome, and remains largely understudied. In this respect, it would be interesting to examine the effects of serine phosphorylation and arginine methylation crosstalk on phase separation, SG recruitment, and the binding to nuclear transport receptors for other (identified) RG/RGG proteins.

We demonstrated that phosphorylation of CIRBP-RGG has profound suppressing effects on its *in vitro* phase separation and SG recruitment (Figure 2 and Figure 7). Phase separation of CIRBP-RGG is induced by the presence of negatively charged RNA, and is driven by multivalent interactions between these oppositely charged biomolecules. The positively charged guanidino group of arginine in the RG/RGG region can be involved in the electrostatic interactions, π-stacking, and hydrogen-bonding with RNA molecules, which promote heterogeneous phase separation (Chong et al., 2018). We propose that the interactions of pCIRBP-RGG with RNA, and hence its RNA-driven LLPS *in vitro*, are reduced via the following mechanisms: 1) addition of phosphate groups to serine residues in the proximity of the RG/RGG repeats decreases the overall charge of the RG/RGG region disfavouring its electrostatic interactions with the phosphate backbone of RNA; 2) the incorporation of phosphate group can alter hydrogen bond network of arginine as phosphates can form strong hydrogen bonds with arginines (Mandell et al., 2007). A recent study reported that SRPK1-phosphorylation of a serine/arginine-rich domain in the nucleocapsid protein of severe acute respiratory syndrome coronavirus 2 (SARS-CoV-2) attenuates its RNA-induced phase separation and partitioning into RNA-rich polymerase-containing condensates (Savastano et al., 2020). Furthermore, Shattuck *et al.* revealed that activity of the yeast kinase Sky1 is required for efficient stress granule disassembly, partly through phosphorylation of Npl3 (Shattuck et al., 2019). These findings suggest that SRPK1-mediated phosphorylation may play a “chaperone-like” role in reducing LLPS of certain substrates and the formation of biomolecular condensates for a larger class of proteins containing low-complexity domains enriched in glycine, serine and positively charged arginine residues. Further investigations are required to clarify the role of phosphorylation on the dynamics of membrane-less organelles in cells.

Additionally, our study revealed that SRPK1-mediated phosphorylation of CIRBP-RGG impairs its binding to TNPO1 (Supplementary Figure S1). The effects of phosphorylation of cargo proteins on binding to their nuclear import receptors seem to be dependent on the system of interest (Nardozzi et al., 2010). As examples of up-regulation of nuclear import upon phosphorylation can serve 1) phosphorylation of Ser385 in the NLS of Epstein-Barr virus nuclear antigen 1 (EBNA-1) protein that increases the binding affinity for an importin α5, which in turn recruits a receptor importin β1 (Kitamura et al., 2006); 2) the RS region of serine/arginine-rich protein ASF/SF2 that acts as the NLS when phosphorylated, while in an unphosphorylated form the protein localizes to the cytoplasm (Lai et al., 2000); or 3) Sky1p-mediated phosphorylation of Npl3p in *S. cerevisiae* which leads to efficient interaction with the nuclear import receptor Mtr10p (Yun and Fu, 2000). Whereas as examples of down-regulation of nuclear import upon phosphorylation can serve: 1) nuclear factor of activated T-cells (NFAT) which resides in the cytoplasm when its serine-rich region is phosphorylated, and translocates to the nucleus upon calcineurin binding that dephosphorylates certain serine residues causing the exposure of the NLS (Ortega-Pérez et al., 2005); or 2) *S. cerevisiae* transcription factor Swi6, in which the presence of phosphoserine160 or phosphomimetic mutation at this site substantially decreases the binding affinity for importin α1, and the nucleocytoplasmic localization and phosphorylation state of Swi6 are dependent on the cell-cycle state (Harreman et al., 2004). Elucidating how nuclear import is regulated is also crucial for a better understanding of the mechanisms governing the onset of neurodegenerative diseases, such as ALS and FTD (Kwiatkowski et al., 2009; Vance et al., 2009). In this respect, arginine methylation has been demonstrated to affect nucleocytoplasmic transport of FUS (Dormann et al., 2012), PABPN1 (Fronz et al., 2011), SERBP1 (Lee et al., 2012), or CIRBP (Aoki et al., 2002). Mutations in the C-terminal NLS of FUS, consisting of a PY-NLS and a RG/RGG region, can lead to reduced binding to TNPO1 and impaired nuclear import (Dormann et al., 2010; Zhang and Chook, 2012). This causes the formation of pathological cytoplasmic FUS aggregates and motor neuron degeneration, with the most severe TNPO1 binding-disrupting mutations resulting in early onset ALS and a particularly fast progression of disease (Dormann et al., 2010). Therefore, understanding the regulation of nuclear import of RG/RGG region-containing proteins by means of PTMs might allow the development of effective therapies against neurodegenerative disorders.

It is intriguing to further speculate about possible advantages of serine phosphorylation for CIRBP and other RG/RGG proteins. Protein phosphorylation by kinases and dephosphorylation by phosphatases provide a dynamic control mechanism critical for the regulation of cellular processes, such as signal transduction, protein synthesis, cell growth, development, division, and aging (Ardito et al., 2017; Gelens and Saurin, 2018). Phosphorylation thus acts as a rapid switch, quickly modulating protein function in response to signals (Hofweber and Dormann, 2019). In contrast, transfer of methyl groups to arginine residues catalysed by protein-arginine methyltransferases is a much slower process (Zhang et al., 2021), and whether this modification can be reversed (and which enzyme catalyses demethylation reaction) remains until now poorly understood (Guccione and Richard, 2019). Arginine methylation is therefore significantly more stable and static compared to serine phosphorylation (Zhang et al., 2021), which can be erased within minutes (Gelens and Saurin, 2018). Consequently, we hypothesize that phosphorylation of CIRBP-RGG offers a means of dynamic regulation of its phase separation *in vitro*, SG association, and protein-protein interactions (e.g. with nuclear import receptor TNPO1) in response to cellular signals. Serine phosphorylation, by suppressing *in vitro* LLPS and triggering disassembly of SGs (i.e. exerts similar effects as arginine methylation), might be beneficial for cells when a rapid modulation of protein function is necessary, or when arginine methylation level is decreased, e.g. due to methionine deprivation, aggregation of PRMTs, or in senescent cells (Hong et al., 2012; Tang et al., 2015; Albrecht et al., 2019). Arginine methylation could then rather serve as a “protein quality control” mechanism regulating protein homeostasis and phase separation, and may be especially relevant in modulating function of neurons that require this modification for a proper stress response (Simandi et al., 2018).

Of note, our findings reveal that CIRBP-RGG can carry both phosphorylation and arginine methylation simultaneously (Figure 6, Supplementary Figure S2). Considering that both modifications play similar roles in regulating phase separation *in vitro* - it remains to be clarified whether they cooperate or interfere with each other. Our bioinformatic analysis suggest that serine phosphorylation within RG/RGG regions might constitute a general mechanism for the dynamic regulation of phase separation of RG/RGG proteins. Still, the manner in which serine phosphorylation affects protein-protein interactions and subcellular localization can be protein specific.

In conclusion, our results imply that PTMs should be seen as key regulators of RBPs phase separation and nucleocytoplasmic transport, and the intricate crosstalk between multiple PTMs serves to fine-tune to changing cellular conditions. As exemplified here for the RG/RGG region of CIRBP, it is essential to study intrinsically disordered regions carrying PTMs when one intends to investigate the regulation of phase separation *in vitro* and the formation of protein aggregates.

## Data Availability

The datasets generated for this study are available on request to the corresponding author. The previous original contributions presented in the study are publicly available. This data can be found here: https://bmrb.io/data_library/summary/index.php?bmrbId=28027.
