# Supplementary material for: Phosphorylation Regulates CIRBP Arginine Methylation, Transportin-1 Binding and Liquid-Liquid Phase Separation
Source: Front Mol Biosci. 2021 Oct 19;8:689687. doi: 10.3389/fmolb.2021.689687 (PMC8562343; doi:10.3389/fmolb.2021.689687)
Supplement: Supplementary file 1 [file DataSheet1.docx]

Supplementary Material

# Supplementary Figures

**Figure S1 – SRPK1-mediated phosphorylation of CIRBP-RGG prevents binding to TNPO1. (A)** Titration of 50 μM CIRBP-RGG into a solution containing 5 μM of TNPO1. The resulting K_d_ yielded 124.4 ± 14.8 nM. The reported errors correspond to the SD of the fit. **(B)** Titration of 50 μM pCIRBP-RGG into a solution containing 5 μM of TNPO1. No detectable binding was observed.


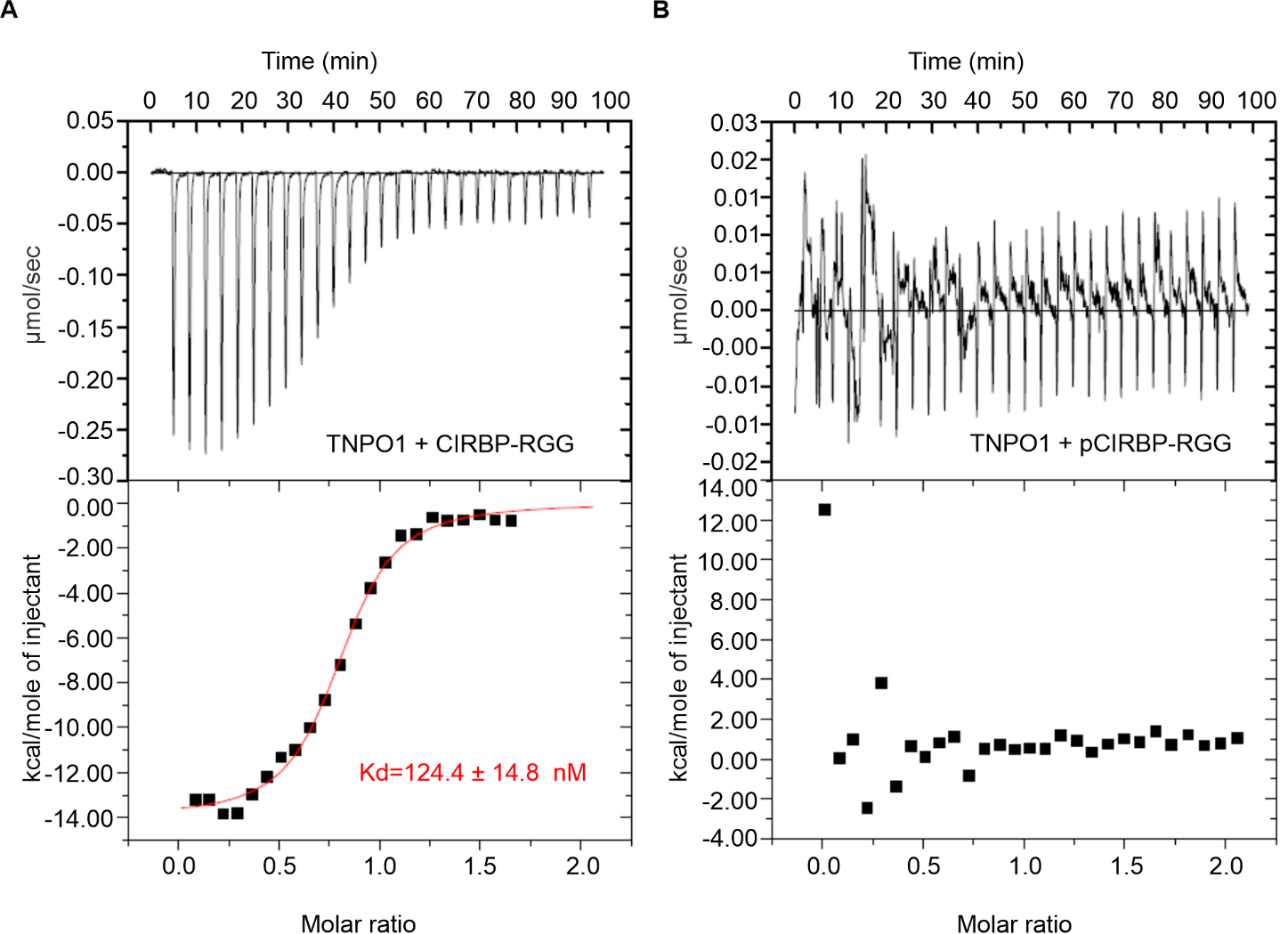


**Figure S2 – Comparison of methylated arginine residues between metCIRBP-RGG and methylated-pCIRBP-RGG.** Strip plots of HCCCONH spectra. ^13^C_δ_ and ^1^H^15^N cross-peaks corresponding to methylated and non-methylated arginines are indicated by dotted lines for (A) metCIRBP-RGG (sample from Fig.6A) and (B) methylated pCIRBP-RGG (sample from Fig.6B).


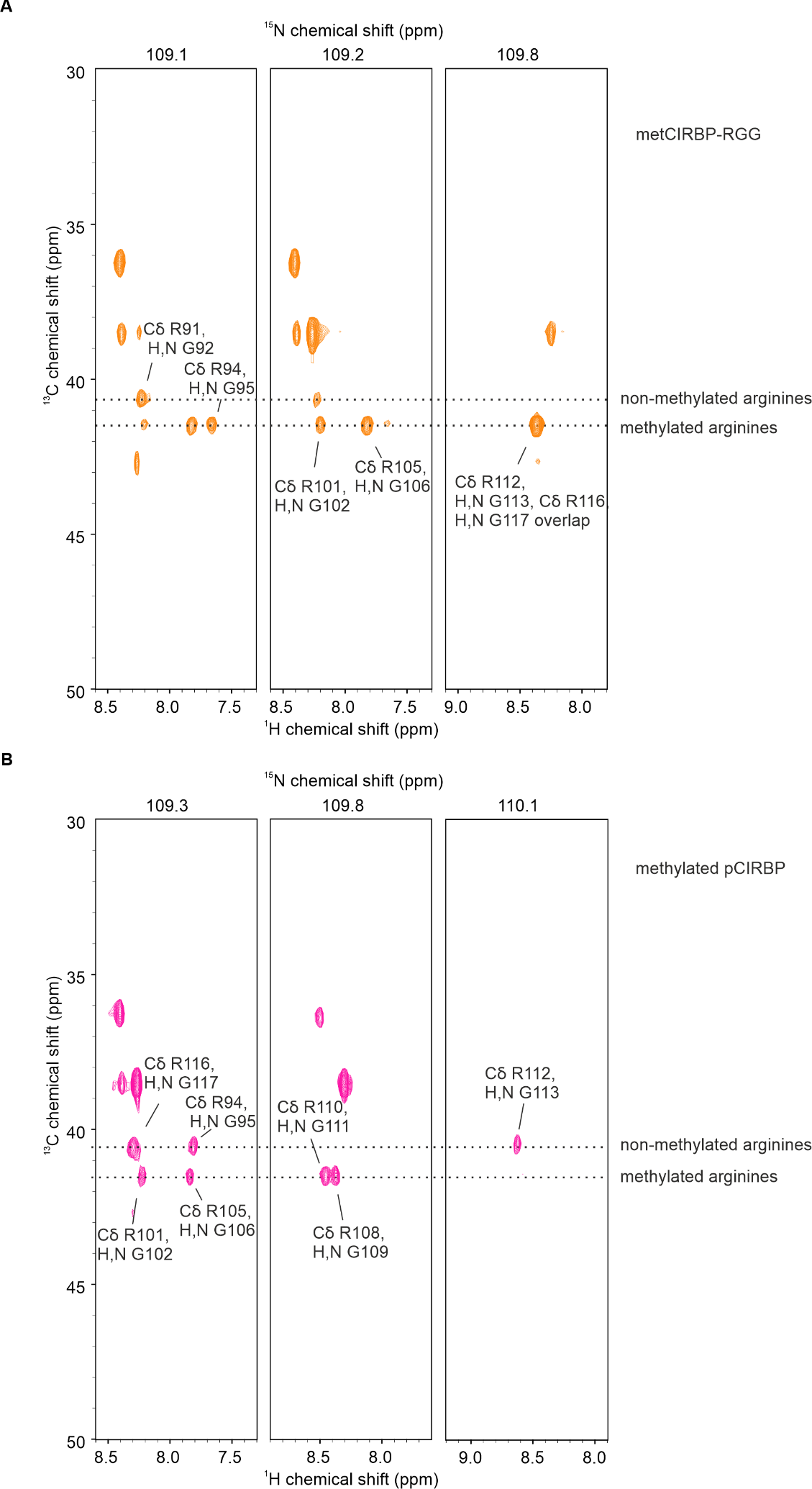


**Dataset S1 – The list of human proteins possessing RS/SR sites within or near to RG/RGG regions.**

| **Number of protein** | **Protein name** |
| --- | --- |
| 1 | Z385C_HUMAN |
| 2 | KIAS3_HUMAN |
| 3 | U2AF5_HUMAN |
| 4 | GA2L1_HUMAN |
| 5 | T121B_HUMAN |
| 6 | CSMD2_HUMAN |
| 7 | PP1RA_HUMAN |
| 8 | GDNF_HUMAN |
| 9 | NIPBL_HUMAN |
| 10 | T53G5_HUMAN |
| 11 | SESN2_HUMAN |
| 12 | PR20D_HUMAN |
| 13 | GCFC2_HUMAN |
| 14 | RBY1E_HUMAN |
| 15 | PAIRB_HUMAN |
| 16 | KCTD8_HUMAN |
| 17 | CHD7_HUMAN |
| 18 | MATN2_HUMAN |
| 19 | RBMX_HUMAN |
| 20 | FILA_HUMAN |
| 21 | STC2_HUMAN |
| 22 | SEM3E_HUMAN |
| 23 | PAPS2_HUMAN |
| 24 | MOBP_HUMAN |
| 25 | SEM3F_HUMAN |
| 26 | PODO_HUMAN |
| 27 | API5_HUMAN |
| 28 | GDF7_HUMAN |
| 29 | KANK3_HUMAN |
| 30 | PGPS1_HUMAN |
| 31 | ZN318_HUMAN |
| 32 | SRSF5_HUMAN |
| 33 | CCD61_HUMAN |
| 34 | ACH10_HUMAN |
| 35 | YLPM1_HUMAN |
| 36 | ACBD4_HUMAN |
| 37 | DLGP3_HUMAN |
| 38 | EIF3A_HUMAN |
| 39 | BTF3_HUMAN |
| 40 | RIMB1_HUMAN |
| 41 | ZSWM8_HUMAN |
| 42 | CO5A3_HUMAN |
| 43 | ZDH24_HUMAN |
| 44 | SPSB2_HUMAN |
| 45 | KRBA1_HUMAN |
| 46 | PCDB8_HUMAN |
| 47 | PAIP1_HUMAN |
| 48 | S35D3_HUMAN |
| 49 | ESPL1_HUMAN |
| 50 | YJ018_HUMAN |
| 51 | TRIPC_HUMAN |
| 52 | CODA1_HUMAN |
| 53 | FUBP3_HUMAN |
| 54 | LAMB2_HUMAN |
| 55 | HNRL2_HUMAN |
| 56 | PRR3_HUMAN |
| 57 | DPYL2_HUMAN |
| 58 | EDEM3_HUMAN |
| 59 | TMED8_HUMAN |
| 60 | RBY1F_HUMAN |
| 61 | RBM3_HUMAN |
| 62 | ZC3H3_HUMAN |
| 63 | HIPL1_HUMAN |
| 64 | RMXL1_HUMAN |
| 65 | MCM4_HUMAN |
| 66 | SHC2_HUMAN |
| 67 | CIRBP_HUMAN |
| 68 | LYRM1_HUMAN |
| 69 | BCL7C_HUMAN |
| 70 | WDR13_HUMAN |
| 71 | CQ102_HUMAN |
| 72 | SORL_HUMAN |
| 73 | CK096_HUMAN |
| 74 | LAR1B_HUMAN |
| 75 | CFA99_HUMAN |
| 76 | WDR33_HUMAN |
| 77 | RREB1_HUMAN |
| 78 | PKHA4_HUMAN |
| 79 | CTDP1_HUMAN |
| 80 | INO80_HUMAN |
| 81 | TSNA1_HUMAN |
| 82 | LS14B_HUMAN |
| 83 | RL3R1_HUMAN |
| 84 | RBY1C_HUMAN |
| 85 | K2C78_HUMAN |
| 86 | CAPR1_HUMAN |
| 87 | MRE11_HUMAN |
| 88 | GREM1_HUMAN |
| 89 | FRAT1_HUMAN |
| 90 | MORN1_HUMAN |
| 91 | MYH7B_HUMAN |
| 92 | DDX43_HUMAN |
| 93 | PCD20_HUMAN |
| 94 | RBM27_HUMAN |
| 95 | BRWD3_HUMAN |
| 96 | IQEC2_HUMAN |
| 97 | EPAB2_HUMAN |
| 98 | ADA2B_HUMAN |
| 99 | KLD7B_HUMAN |
| 100 | SRSF1_HUMAN |
| 101 | GGYF2_HUMAN |
| 102 | CO6A5_HUMAN |
| 103 | ZSA5C_HUMAN |
| 104 | ADCY5_HUMAN |
| 105 | PPN_HUMAN |
| 106 | K2C1B_HUMAN |
| 107 | SPRN_HUMAN |
| 108 | DDX54_HUMAN |
| 109 | RBM6_HUMAN |
| 110 | SCNNA_HUMAN |
| 111 | FBX15_HUMAN |
| 112 | IF4G1_HUMAN |
| 113 | CABP4_HUMAN |
| 114 | COQ5_HUMAN |
| 115 | F120C_HUMAN |
| 116 | FBCD1_HUMAN |
| 117 | ZN598_HUMAN |
| 118 | SRRM5_HUMAN |
| 119 | SRSF4_HUMAN |
| 120 | RM41_HUMAN |
| 121 | ZBT7A_HUMAN |
| 122 | AGAP2_HUMAN |
| 123 | HNRPK_HUMAN |
| 124 | ROBO3_HUMAN |
| 125 | GNL1_HUMAN |
| 126 | DDX5_HUMAN |
| 127 | MYO15_HUMAN |
| 128 | RBY1D_HUMAN |
| 129 | ZC3H4_HUMAN |
| 130 | STAG1_HUMAN |
| 131 | CORA1_HUMAN |
| 132 | TMC6_HUMAN |
| 133 | HXC11_HUMAN |
| 134 | ANK1_HUMAN |
| 135 | MBD1_HUMAN |
| 136 | KMT2C_HUMAN |
| 137 | RBM26_HUMAN |
| 138 | SH2B1_HUMAN |
| 139 | HNRPQ_HUMAN |
| 140 | PR20A_HUMAN |
| 141 | TR150_HUMAN |
| 142 | CC080_HUMAN |
| 143 | DHX9_HUMAN |
| 144 | TGM1_HUMAN |
| 145 | SHAN1_HUMAN |
| 146 | IF4B_HUMAN |
| 147 | SPEG_HUMAN |
| 148 | SNUT2_HUMAN |
| 149 | IG2AS_HUMAN |
| 150 | MY15B_HUMAN |
| 151 | RNC_HUMAN |
| 152 | ING5_HUMAN |
| 153 | PRD10_HUMAN |
| 154 | ATL4_HUMAN |
| 155 | AR6P4_HUMAN |
| 156 | SNUT1_HUMAN |
| 157 | CTGE2_HUMAN |
| 158 | BPTF_HUMAN |
| 159 | EYA1_HUMAN |
| 160 | CCD9B_HUMAN |
| 161 | COBA1_HUMAN |
| 162 | KHDR3_HUMAN |
| 163 | M3K13_HUMAN |
| 164 | H2BFM_HUMAN |
| 165 | FXR1_HUMAN |
| 166 | TRI67_HUMAN |
| 167 | TMCO3_HUMAN |
| 168 | SAMD5_HUMAN |
| 169 | ZN608_HUMAN |
| 170 | SRRM2_HUMAN |
| 171 | ESYT2_HUMAN |
| 172 | LOXH1_HUMAN |
| 173 | RBP56_HUMAN |
| 174 | RBY1B_HUMAN |
| 175 | TM271_HUMAN |
| 176 | IF2B3_HUMAN |
| 177 | DHX57_HUMAN |
| 178 | MZF1_HUMAN |
| 179 | PKHG3_HUMAN |
| 180 | RBBP6_HUMAN |
| 181 | MMP21_HUMAN |
| 182 | RN169_HUMAN |
| 183 | SSF1_HUMAN |
| 184 | SCRIB_HUMAN |
| 185 | CAC1I_HUMAN |
| 186 | CWC22_HUMAN |
| 187 | APCD1_HUMAN |
| 188 | TF3C1_HUMAN |
| 189 | UBP2L_HUMAN |
| 190 | YTDC2_HUMAN |
| 191 | DYRK2_HUMAN |
| 192 | DCAF6_HUMAN |
| 193 | SFPQ_HUMAN |
| 194 | CAPR2_HUMAN |
| 195 | DDX3Y_HUMAN |
| 196 | KRT85_HUMAN |
| 197 | TUSC1_HUMAN |
| 198 | ARHG5_HUMAN |
| 199 | U2AF1_HUMAN |
| 200 | BCLF1_HUMAN |
| 201 | CA229_HUMAN |
| 202 | CAC1B_HUMAN |
| 203 | PABP2_HUMAN |
| 204 | SNIP1_HUMAN |
| 205 | AF10_HUMAN |
| 206 | ZC3H1_HUMAN |
| 207 | PININ_HUMAN |
| 208 | TNIP2_HUMAN |
| 209 | RBY1A_HUMAN |
| 210 | FBN2_HUMAN |
| 211 | CXL17_HUMAN |
| 212 | CC185_HUMAN |
| 213 | HNRPR_HUMAN |
| 214 | SETX_HUMAN |
| 215 | DYRK3_HUMAN |
| 216 | AVEN_HUMAN |
| 217 | YQ037_HUMAN |
| 218 | ANK3_HUMAN |
| 219 | TM214_HUMAN |
| 220 | LCAP_HUMAN |
| 221 | IRS4_HUMAN |
| 222 | MET14_HUMAN |
| 223 | PAPS1_HUMAN |
| 224 | FMR1_HUMAN |
| 225 | HERC6_HUMAN |
| 226 | PR20C_HUMAN |
| 227 | DNJB6_HUMAN |
| 228 | PRC2A_HUMAN |
| 229 | WDR11_HUMAN |
| 230 | ZBTB3_HUMAN |
| 231 | PRC2C_HUMAN |
| 232 | SYNE3_HUMAN |
| 233 | ID3_HUMAN |
| 234 | VSX1_HUMAN |
| 235 | R3HD4_HUMAN |
| 236 | EMC4_HUMAN |
| 237 | BMP1_HUMAN |
| 238 | MIS_HUMAN |
| 239 | TRIM9_HUMAN |
| 240 | EDN3_HUMAN |
| 241 | M3K20_HUMAN |
| 242 | C170B_HUMAN |
| 243 | CSPG4_HUMAN |
| 244 | DDX21_HUMAN |
| 245 | RBM33_HUMAN |
| 246 | U2AFM_HUMAN |
| 247 | PCDBD_HUMAN |
| 248 | EWS_HUMAN |
| 249 | CT202_HUMAN |
| 250 | TENX_HUMAN |
| 251 | RMXL2_HUMAN |
| 252 | TRNK1_HUMAN |
| 253 | PR20B_HUMAN |
| 254 | RPC7L_HUMAN |
| 255 | CAHM3_HUMAN |
| 256 | CIP4_HUMAN |
| 257 | SLTM_HUMAN |
| 258 | ZFP91_HUMAN |
| 259 | OSBL9_HUMAN |
| 260 | TM275_HUMAN |
| 261 | IF4H_HUMAN |
| 262 | CDAN1_HUMAN |
| 263 | CONA1_HUMAN |
| 264 | ROA1_HUMAN |
| 265 | NCAS2_HUMAN |
| 266 | NKTR_HUMAN |
| 267 | ZO2_HUMAN |
| 268 | KHDR1_HUMAN |
| 269 | VTNC_HUMAN |
| 270 | CNKR3_HUMAN |
| 271 | CELR2_HUMAN |
| 272 | ROA0_HUMAN |
| 273 | ILF2_HUMAN |
| 274 | MAST3_HUMAN |
| 275 | ITIH5_HUMAN |
| 276 | RHG33_HUMAN |
| 277 | SP16H_HUMAN |
| 278 | SRSF8_HUMAN |
| 279 | FBX33_HUMAN |
| 280 | E41LB_HUMAN |
| 281 | THOC4_HUMAN |
| 282 | TNF12_HUMAN |
| 283 | KMT2B_HUMAN |
| 284 | RSRC1_HUMAN |
| 285 | KDM4E_HUMAN |
| 286 | PLXB2_HUMAN |
| 287 | SPIDR_HUMAN |
| 288 | LRFN2_HUMAN |
| 289 | SEM3G_HUMAN |
| 290 | FGF2_HUMAN |
| 291 | M14OS_HUMAN |
| 292 | ZN469_HUMAN |
| 293 | GGYF1_HUMAN |
| 294 | K1614_HUMAN |
| 295 | OTUD4_HUMAN |
| 296 | LARP6_HUMAN |
| 297 | HECD1_HUMAN |
| 298 | SOX5_HUMAN |
| 299 | SMTN_HUMAN |
| 300 | SPB1_HUMAN |
| 301 | VGFR3_HUMAN |
| 302 | SCAFB_HUMAN |
| 303 | EYA4_HUMAN |
| 304 | CNNM2_HUMAN |
| 305 | CC88B_HUMAN |
| 306 | PRC2B_HUMAN |
| 307 | ABHGA_HUMAN |
| 308 | PQBP1_HUMAN |
| 309 | STRP2_HUMAN |
| 310 | DDX3X_HUMAN |
| 311 | PM2P1_HUMAN |
| 312 | WIPF2_HUMAN |
| 313 | PITM1_HUMAN |
| 314 | K2C71_HUMAN |
| 315 | DSRAD_HUMAN |
| 316 | MEGF8_HUMAN |
| 317 | PAGE4_HUMAN |
| 318 | CROCC_HUMAN |
| 319 | NFXL1_HUMAN |
| 320 | GABT_HUMAN |
| 321 | CCBE1_HUMAN |
| 322 | MHAS1_HUMAN |
| 323 | TP53B_HUMAN |
| 324 | F120A_HUMAN |
| 325 | TR13C_HUMAN |
| 326 | APOL5_HUMAN |
| 327 | F1712_HUMAN |
| 328 | 3BP5L_HUMAN |
| 329 | PR20E_HUMAN |
| 330 | TDRD3_HUMAN |
| 331 | MAGIX_HUMAN |
| 332 | SC16B_HUMAN |
| 333 | SEM4G_HUMAN |
| 334 | WWC3_HUMAN |
| 335 | CSTFT_HUMAN |
| 336 | CNBP_HUMAN |
| 337 | SRRT_HUMAN |
| 338 | RPA2_HUMAN |

**Dataset 2 – The list of identified human proteins containing phosphorylation and/or arginine methylation sites within RG/RGG regions with RS/SR site.**

Phosphorylation sites (serine, threonine, tyrosine) are indicated in red, and arginine methylation sites are shown in blue.

| **Protein name** | **Serine phosphorylation sites** | **Amino acids sequence** | **Arginine methylation sites** |
| --- | --- | --- | --- |
| **Proteins possessing arginine methylation and serine phosphorylation sites** | | | |
| PRC2A_HUMAN | S1056 | CRGRGRGE YFARGRGFRG TYGGRGRGAR SREFRSYREF RGDDGRGGGT | R1024/26/28/1034/36/39&156 |
| RBMX_HUMAN | S380 | RGYPPP RDSYSSSSRG APRGGGRGGS RSDRGGGRSR Y | R355/360/69/73/77/81 |
| EIF3A_HUMAN | S1059/1149 | DRGPRR GMDDDRGPRR GGADDERSSW RNADDDRGPR RGLDDDRGPR RGMDDDRGPR RGMDDDRGPR RGMDDDRGPR RGLDDDRGPW RNADDDRIPR RGAEDDRGPW RNMDDDRLSR | R1057/1071/1111/1117/1127/1137 |
| PAIRB_HUMAN | S187 | IRGRGGLGR GRGGRGRGMG RGDGFDSRGK R | R163 /65/75/77/80/88 |
| MRE11_HUMAN | S590 | KGR GRGRGRRGGR GQNSASRGGS QRGR | R570/72/74/76/77/80/87/92/94 |
| KMT2B_HUMAN | S286 | R RGGQSSRGGR GGRGRGRGGG | R280/93/95/97 |
| ILF2_HUMAN | S15 | MRGDRGRGRG GRFGSRGGPG GGFR | R5/7/9/16/24 |
| CSTFT_HUMAN | S466/467 | TRGMEA RGMDARGLEM RGPVPSSRGP | R461 |
| HNRPQ_HUMAN | S509/524/531 | ARGRGG RGARGAAPSR GRGAAPPRGR AGYSQRGGPG SARGVRGARG GAQQQRGRGV RGARGGRGGN | R496/98/501/4/20/26/33 |
| LAR1B_HUMAN | S135/136/139 | SSVRSE GGNIRGSFRG RGRGRGRGRG RGRGNPR | R138/45/53/55 |
| ROA0_HUMAN | S188 | GSRS SRGGRGGRGR GGGR | R189/92/95/98/190/94 |
| THOC4_HUMAN | S18 | NRSQR GGRGGGRGRG RAGSQGGRGG G | R31/38 |
| GGYF2_HUMAN | S139 | LTGRGGG GTVVGAPRGR SSSRGRGRGR GECGFYQRSF DEVEGVFGRG GGR | R107/118/120/149/153 |
| BCL7C_HUMAN | S69 | RSR GRERRGRGAS PRGG | R77/82 |
| RBM33_HUMAN | S273 | GR YSSRRGGRRG GPLMCRGVGD QRRESTERGR | R279/286/298 |
| BCLF1_HUMAN | S54 | YSRDYRRD YRNNRGMRRP YGYRGRGRGY Y | R55/76/78 |
| UBP2L_HUMAN | S130 | Y SRRRGGPPRR GRGASRGREF RGQENGLDGT KSGGPSGRGT ERGRRGRGRG RGGSGRRGGR F | R151/168/172/174/187/190 |
| LS14B_HUMAN | S349 | SG RFLRGRSSRG GFRGGRGNGT | R351/354/356 |
| ZN608_HUMAN | S454/473 | ESRGLQNK NRGGANGKGR RGSLNASGRR | R473 |
| **Proteins possessing phosphorylation sites without arginine methylation sites within RG/RGG regions with RS/SR site** | | | |
| ZN608_HUMAN | S440 | EMRG GRGRGKRARS A |  |
| SPRN_HUMAN | S47 | GGRGG ARGSARGGVR GGARGASR | - |
| KDM4E_HUMAN | S452 | GSGRG RGRGQGQGRG CSRGRGHG | - |
| **Proteins possessing phosphorylation sites (not only at serines) and arginine methylation sites** | | | |
| RBP56_HUMAN | - | DRGG YGGDRGGYGG DRGGYGGDRG GYGGDRSRGG YGGDRGGGSG | R498/505/512/518/528/535 |
| PRC2C_HUMAN | - | YRGRGR GEYYSRGRSY RGSYGGRGRG GRGHTRD | R1186/88/1190/96/98 |
| TR150_HUMAN | S823 | SRLGTKDFV GPSERGGGRA RGTFQFRARG RGWGRGNYSG | R825/829/831/37/39/41/45 |
| KHDR1_HUMAN |  | SRGRGVPVRG RGAAPPPPPV PRGRGVGPPR GALVRGTPVR GAITRGATVT RGVP | R282/84/89/91/302/304/310/315/320/325 |
| **Proteins without reported phosphorylation sites, and with/without arginine methylation sites** | | | |
| ZC3H1_HUMAN | - | RFRFRGRP YRGGSRWSRG RGVGERGGKP | - |
| PRD10_HUMAN | - | VFSRTRGR GRGRGKRRFG PGRRPGRPPK | - |
| SHC2_HUMAN | - | SRCR GAGSRGSRGG RGAA | - |
| CK096_HUMAN | - | LRGRG FSRNPRGRGL PSGAGWRGAG GAGEGAVTFP ERRGD | - |
| SCAFB_HUMAN | - | N RGRGRGNRGR GTYRSS | R1084 |
| API5_HUMAN | - | QR GAFRGSRGGR GWGTRGNRSR GRLY | R500/4/7/10 |
| MET14_HUMAN | - | SKSDRGG GAPRGGGRGG TSAGRGRERN RSNFRGERGG FRGGRGGAHR | - |
| BRWD3_HUMAN | - | TRGT GGRGRWGRWG RWSRGGRGRG GRGRGSRGRG GGGTRGRGRG RGGRGASRGA T | - |
| AVEN_HUMAN | - | GR RGRGRGRGFR GARGGRGGGG APRGSRR | R50 |
| EWS_HUMAN | - | R GRGRGGFDRG GMSRGGRGGG RGGMGSA --//-- R GGDRGGFPPR GPRGSRGNPS GG | R300/2/4/9/14/17/21--//-- R490/94/80/83/86 |
| DDX54_HUMAN | - | R RGPERRGGKR DRGQGASR | R802 |
| TP53B_HUMAN | - | PRGRGR RGRPPSRTTG | R1396/98/1400/01/03 |
| RBM26_HUMAN | - | KRGI LSSGRGRGIH SRGRGAVHGR GRGRGRGRGV | R855/57/78 |
| GGYF1_HUMAN | - | SRGRGS TRSRGRGRGD SC | - |
| CNBP_HUMAN | - | GRGRGMR SRGRGGFTSD RGF | R25/27/30/32/34/41 |
